# Mismatch Responses to Prosodic Changes in Children With Developmental Language Disorder: A Longitudinal Approach

**DOI:** 10.1111/ejn.70710

**Published:** 2026-10-12

**Authors:** Liis Themas, Gesa Schaadt, Pärtel Lippus, Marika Padrik, Liis Kask, Ulvi Vaher, Mairi Männamaa, Kairi Kreegipuu

**Affiliations:** ^1^ Institute of Estonian and General Linguistics University of Tartu Tartu Estonia; ^2^ Institute of Psychology University of Tartu Tartu Estonia; ^3^ Department of Education and Psychology Freie Universität Berlin Berlin Germany; ^4^ Department of Neuropsychology Max Planck Insitute of Human Cognitive and Brain Sciences Leipzig Germany; ^5^ Institute of Educational Science University of Tartu Tartu Estonia; ^6^ Department of Radiology, Institute of Clinical Medicine University of Tartu Tartu Estonia; ^7^ Childrens Clinic Tartu University Hospital Tartu Estonia

**Keywords:** child development, developmental language impairment, EEG, longitudinal study, MMN, prosody

## Abstract

Developmental language disorder is characterized by difficulties in auditory processing and prosody discrimination. Estonian word prosody features a unique three‐way quantity distinction, characterized by changes in syllable duration and pitch contour, which differentiate word meaning and grammatical cases. In this study, we examined the Mismatch Responses longitudinally in 23 children with developmental language disorder and 25 age and biological sex matched typically developing peers (age at first measurement 4;6–6;5 years; age at second measurement 5;6–7;5 years). A multifeature paradigm was used in which all stimuli were meaningful words; two deviants differed from the standard, one in syllable duration and the other in both syllable duration and pitch contour. We also tested the discrimination of these prosodic features with behavioral tasks at both measurements. For duration discrimination, an early positive mismatch response diminished from first to second measurement, while a subsequent negative mismatch response increased. Significant longitudinal changes were observed in the typically developing group but not in children with developmental language disorder, while direct comparisons of maturational trajectories did not reveal significant group differences. For the duration and pitch contrast, in both groups and at both assessments, no mismatch response was detected. Additionally, no association was observed at the individual level between maturational changes in mismatch responses and behavioral discrimination at either measurement. These findings provide insights into the maturation of prosodic change detection processes and contribute to a better understanding of developmental language disorder and its manifestations.

## Graphical abstract

This longitudinal study examined the maturation of mismatch responses to prosodic changes in preschool children with and without developmental language disorder. For duration discrimination, an early positive mismatch response diminished while a subsequent negative mismatch response increased, with significant longitudinal changes observed only in typically developing children. No mismatch response was detected for the combined duration and pitch discrimination at either assessment, and no association was observed between mismatch responses and behavioral discrimination.

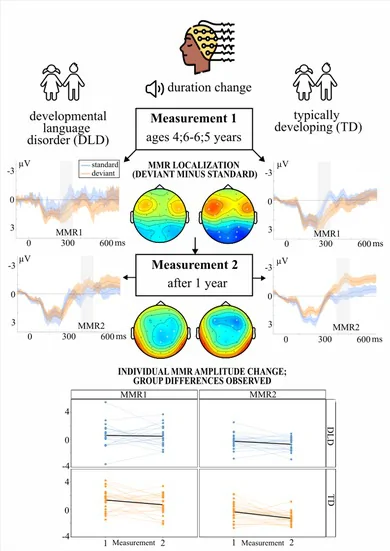

**Abbreviations**
CI: confidence intervals
*d*′: sensitivity index
DD: developmental dyslexia
*df*
: degrees of freedom
DLD: developmental language disorder
durQ1: deviant stimulus in the first quantity degree differing from the standard in syllable duration
EEG: electroencephalography
ERPs: event‐related potentials
imp.: imperative
inf.: infinitive form
ISI: interstimulus interval
IQ: intelligence quotient
LDN: late discriminative negativity
LMM: linear mixed model
M: mean
MADE: the Maryland Analysis of Developmental EEG
*max*
: maximum value
*min*
: minimum value
MMN: mismatch negativity
MMR: mismatch response
*N*
: the number of observations
NMAR: not missing at random
nom.: nominative case
*p*
: probability value
pitchdurQ3: deviant stimulus in the third quantity degree, differing from the standard in syllable duration and pitch contour
Q1: first quantity degree
Q2: second quantity degree
Q3: third quantity degree
*r*
: Pearson correlation coefficient
RT: reaction time
*R* ^2^
: *R* squared
SD: standard deviation
sg: singular
sg2: second person singular
*t*
: a ratio of the estimated fixed effect to its standard error
T1: first assessment
T2: second assessment after 1 year
TD: typically developing
TW1: first time window
TW2: second time window
*W*
: Wilcoxon test statistic
WPPSI‐IV UK: Wechsler Preschool and Primary Scale of Intelligence, fourth edition, United Kingdom version
*χ* ^2^
: chi‐squared statistic
μV: microvolts

## 1 Introduction

### 1.1 Developmental Language Disorder

Developmental language disorder (DLD) emerges during development and is characterized by persistent difficulties in acquiring and using language across different domains, such as phonology, vocabulary, and morphology (Bishop et al. 2017; Gillam et al. 2021), independent of intellectual disabilities, hearing impairments, or primary physical or neurological disorders (Bishop et al. 2017). One of these children's core deficits is an atypical auditory processing (Kujala and Leminen 2017; Näätänen et al. 2014), including prosody perception (Calet et al. 2021; Cumming et al. 2015; Leonard and Kueser 2019; Richards and Goswami 2019; Sundström et al. 2019).

### 1.2 The Estonian Three‐Way Quantity Distinction

Prosody refers to phonetic phenomena that operate at the suprasegmental level, that is, extending over more than one speech sound (Asu et al. 2016). Estonian is one of several quantity languages where phoneme length, as a prosodic feature, plays a crucial role in differentiating between word meanings and grammatical forms. Both vowels and consonants in the stressed syllable exhibit a three‐way length distinction: They can be short (quantity 1—Q1; e.g., [sɑtɑ], [kɑlɑ]), long (quantity 2—Q2; e.g., [sɑːtɑ], [kɑllɑ]) or overlong (quantity 3—Q3; e.g., [sɑːːtɑ], [kɑlːlɑ]; Lippus et al. 2013). Quantity distinction is marked primarily by the duration of the stressed initial syllable, which is longer in the case of higher degrees of quantity, while the following unstressed syllable is compensatorily shortened. In addition, the quantity is marked with the fundamental frequency contour: In Q1 and Q2, the pitch fall is aligned with the end of the stressed syllable, whereas in Q3, the fall takes place in the first half of the stressed syllable (Lippus et al. 2013). To date, only one study (Themas, Nickel, et al. 2025) has been conducted on auditory quantity perception with three‐way quantity distinction in Estonian children.

### 1.3 Auditory Discrimination and Its Maturation

#### 1.3.1 Measuring the Ability of Auditory Discrimination

Auditory perception across maturation can be objectively and noninvasively investigated with electroencephalography (EEG) using event‐related potentials (ERPs). An ERP reflecting the brain's ability to discriminate different characteristics of speech, such as different quantity degrees, is the mismatch negativity (MMN), a well‐studied fronto‐central negativity typically elicited 100–200 ms after the onset of a deviating stimulus within a stream of standard stimuli (Näätänen et al. 1978). The MMN is typically measured with an oddball paradigm (Squires et al. 1975) but can also be elicited with the multifeature paradigm (Optimum‐1; Näätänen et al. 2004), in which more than one deviant stimulus can be tested, such as different quantity degrees in the Estonian language.

In studies investigating children, the MMN is often referred to as the mismatch response (MMR) because developmental studies report positive polarities in younger individuals, such as infants (Chen et al. 2022; Cheng et al. 2013; Cheng et al. 2015; Cheng and Lee 2018; Friederici et al. 2002; He et al. 2007; Leppänen et al. 2004; Mueller et al. 2012; Virtala et al. 2022; Werwach et al. 2022) and small children (ages 3–8 years; Campos et al. 2023; Chen et al. 2016; Cheng et al. 2021; Lee et al. 2012; Liu et al. 2014; Nan et al. 2018; Partanen, Torppa, et al. 2013). Evidence suggests that while children's auditory processing matures, the positive MMR decreases and/or reverses polarity and becomes a negative adult‐like MMN (Chen et al. 2022; Cheng et al. 2013; Cheng et al. 2015; Choudhury and Benasich 2011; Lee et al. 2012; Maurer et al. 2003; Morr et al. 2002; Niemitalo‐Haapola et al. 2017; Partanen, Torppa, et al. 2013; Putkinen et al. 2019; Schaadt et al. 2015; Shafer et al. 2010; Themas et al. 2023; Werwach et al. 2022; Yu et al. 2019). The different MMR polarities are discussed to reflect distinct underlying neural processes (Rivera‐Gaxiola et al. 2005), potentially linked to bottom‐up (positive MMR) and top‐down (negative MMR) processing pathways, which are thought to involve separate neural generators (see Schaadt et al. 2015 for similar argumentation).

Next to polarity, the latency of children's MMRs (both positive and negative) changes with maturation. Compared to adults, the latencies of the MMRs in children are delayed and decrease with age, which has been discussed to indicate that children's auditory discrimination processes are less automated compared to those of adults (Choudhury and Benasich 2011; Liu et al. 2014; Morr et al. 2002; Shafer et al. 2000; Shafer et al. 2010).

In some studies, the positive MMR has been found to be followed by an ERP of negative polarity. Some suggest it to be a coexisting negative MMR (Lee et al. 2012; Liu et al. 2014; Morr et al. 2002; Partanen, Torppa, et al. 2013; Shafer et al. 2010), while others interpreted this negativity to be the late discriminative negativity (LDN). LDN is located fronto‐centrally and appears 350–600 ms or even later after deviance onset (Čeponienė et al. 2004; Korpilahti et al. 1995; Linnavalli et al. 2018; Putkinen et al. 2012). Compared to the MMR, the LDN has different neural generators (Čeponienė et al. 2004; Hommet et al. 2009), and its function and maturational trajectory are ambiguous. Some studies indicate that the LDN is modulated by the deviance complexity showing larger values for more difficult changes reflecting additional processing of the deviance (Bishop et al. 2011; David et al. 2020). The LDN's polarity and latency were also shown to be modulated by maturation. Some studies found a decrease in amplitude (Bishop et al. 2011; Hommet et al. 2009; Linnavalli et al. 2018; Liu et al. 2014; Morales et al. 2022) and decrease in latency with age (Bishop et al. 2011; Mueller et al. 2008), while others report no latency change (David et al. 2020; Hommet et al. 2009).

#### 1.3.2 Auditory Discrimination of Duration and Pitch

MMRs have been widely used to investigate children's discrimination of speech sounds, including both segmental contrasts and suprasegmental features. Because Estonian quantity is a linguistically contrastive feature, its perception relies on the discrimination of prosodic cues, namely, duration and pitch contour, which in turn support lexical interpretation. For clarity, we therefore first review MMR studies addressing the discrimination of these acoustic‐prosodic features and then consider evidence from word‐level paradigms illustrating how such cues contribute to lexical distinctions. This structure reflects two complementary levels of analysis rather than implying a separation between prosodic and lexical processing.

The discrimination of duration and pitch emerges early in life and undergoes maturational changes through childhood, as reflected by the MMR becoming more negative and exhibiting shorter latency with increasing age (Friederici et al. 2002; Friedrich et al. 2004; He et al. 2007; Polver et al. 2023; Virtala et al. 2022; Werwach et al. 2022) including during preschool and school age (Chobert et al. 2014; Linnavalli et al. 2018; Shafer et al. 2000).

Conclusions can also be drawn from studies on Mandarin lexical tone perception using linguistic stimuli, also requiring the perception of pitch contour information (e.g., Wu et al. 2025). Perception of lexical tone differences emerges in early infancy (Chen et al. 2022; Cheng et al. 2013; Cheng et al. 2015; Cheng and Lee 2018) and continues to mature through preschool ages and adolescence (Chen et al. 2016; Cheng et al. 2021; Lee et al. 2012; Liu et al. 2014; Wu et al. 2025).

Unlike in Mandarin lexical tone perception, where pitch contour serves as the primary cue, listeners of Estonian must additionally rely on duration to distinguish between Q2 and Q3. In this case, the pitch contrast emerges gradually from the beginning of the word and is followed by the durational contrast. Adult studies have shown that in some cases the presence of multiple differences between the standard and deviant stimuli modulates the MMN. Deviants differing by two features from the standard elicit a stronger MMN compared to deviants differing by one feature (Paavilainen et al. 2001; Ylinen et al. 2005). Nevertheless, in our previous study (Themas, Nickel, et al. 2025), we found no significant MMR in children discriminating combined pitch contour and durational differences (Q2 vs. Q3) in meaningful words. In contrast, Lyu et al. (2024) reported significant MMNs in Estonian adults during discrimination of combined pitch contour and duration changes in meaningful Q2 and Q3 words, but no enhanced MMN due to multiple differences between the standard and the deviant.

#### 1.3.3 Auditory Discrimination of Meaningful Words

In addition to indexing the ability to discriminate prosodic features, the MMN has also been shown to reflect lexical processing in adults (Aleksandrov et al. 2017; Hasting et al. 2008; Pettigrew et al. 2004; Pulvermüller et al. 2001; Zora et al. 2015; Zora et al. 2016; Zora et al. 2023). Prosodic features appear to activate memory‐based predictions about word meaning, as indicated by enhanced MMN amplitudes and shorter latencies for words compared to pseudowords with similar prosody (Pulvermüller et al. 2001; Zora et al. 2015; Zora et al. 2016; Zora et al. 2023). There is also evidence of higher sensitivity to prosodic changes in meaningful words as the brain leverages prior lexical knowledge alongside the incoming acoustic information (Zora et al. 2015). One might also assume that in words with a high frequency in a language, the processing of prosodic features is more facilitated. This assumption is consistent with the view that differences between high‐frequency and low‐frequency words resemble those observed between high‐frequency words and pseudowords. Furthermore, high‐frequency words are processed faster and more automatically (Alexandrov et al. 2011; Aleksandrov et al. 2017).

In studies involving children, evidence is scarce. Strotseva‐Feinschmidt et al. (2015) found a more mature MMR in response to high frequency function words presented as deviants compared to similarly presented low frequency function words in 3‐year‐olds. Campos et al. (2023) found more robust MMNs in adults when differentiating meaningful words compared to nonwords but did not observe this effect in 4‐ to 5‐year‐old children. Furthermore, they found positive MMRs in children in the nonword condition, but negative MMRs in the meaningful word condition, suggesting a more mature processing of meaningful words compared to nonwords. However, they did not find any processing differences between TD children and children with DLD (Campos et al. 2023).

#### 1.3.4 Auditory Discrimination Abilities in Children With DLD

In contrast to the findings reported above, which did not reveal differences between TD children and children with DLD (Campos et al. 2023), other studies have found evidence of brain‐level differences in auditory processing between these groups. Even in infancy, children at risk of language impairment display reduced or delayed MMRs compared to TD participants in response to different auditory contrasts, including duration and pitch discrimination (Benasich et al. 2006; Cantiani et al. 2016; Choudhury and Benasich 2011; Friedrich et al. 2004; Riva et al. 2018; Weber et al. 2005).

Much research has been conducted on studying perception of pitch changes in nonlinguistic tone stimuli, where preschool and school‐aged children with DLD showed attenuated MMN/MMR amplitudes compared to TD children (for review see Bishop 2007 and Kujala and Leminen 2017). Group differences between children with DLD and TD of a similar age to the current sample concerning their MMRs to lexical tones were also found, such that children with DLD show less prominent or less mature MMRs (Y. Chen et al. 2016; Cheng et al. 2021). Few studies have researched duration perception in DLD, and the one investigating participants with comparable age to the current study found no group differences in MMNs in response to duration changes of nonlinguistic tone stimuli (Uwer et al. 2002). Research on children with developmental dyslexia (DD) using linguistic stimuli can provide additional insights into expected group differences. DLD and DD seem to stem from similar language processing difficulties (Leonard 2014) and studies on duration change detection in school‐aged children (older than the current sample) with DD showed reduced negative MMR amplitudes compared to typically developing (TD) children (Männel et al. 2017; Schaadt and Männel 2019).

There is also limited research investigating the maturation of brain‐level differences in auditory perception, as reflected in the MMR, either longitudinally or by age group comparisons in the DLD population (Bishop et al. 2010; Y. Chen et al. 2016; Choudhury and Benasich 2011). To better understand DLD and its manifestations, it is, however, crucial not only to measure auditory processing at a single time point, but to also observe its maturation, as DLD itself evolves over time. Longitudinal studies offer valuable insights into several key aspects of DLD. The MMR measured at infancy and toddlerhood has been shown to be predictive of future language abilities (Cantiani et al. 2016; Chen et al. 2016; Choudhury and Benasich 2011; Friedrich et al. 2009; Guttorm et al. 2005; Schaadt et al. 2015; Schaadt et al. 2023; Weber et al. 2005; Zhao et al. 2021) and therefore could also be suitable for predicting future outcomes of DLD at later ages.

### 1.4 Relationship Between Neurophysiological and Behavioral Findings of Auditory Discrimination

The association between MMR and later language abilities suggests the MMR to be indicative of behavioral auditory discrimination. However, there is very limited evidence on an association between the MMR and behavioral auditory discrimination abilities and only a few studies have specifically investigated this association in healthy individuals and even less so in individuals with DLD. In healthy individuals, some studies have found significant associations between some neurophysiological and behavioral measures of auditory discrimination (McArthur and Bishop 2005; Nan et al. 2018; Pihko et al. 2006), while others have not (Bishop et al. 2010; Halliday et al. 2014; McArthur and Bishop 2005; Nan et al. 2018; Pihko et al. 2006; Uwer et al. 2002). These contradictory findings might be explained by different task designs of behavioral auditory discrimination tasks and type of contrast tested in the MMR paradigm (see, e.g., Pakarinen et al. 2007), but it could also indicate that different partially independent mechanisms are tested by neurophysiological (preattentive) and behavioral (attentive, conscious) auditory discrimination paradigms. Future studies should specifically test the association between neurophysiological and behavioral auditory discrimination processes. Depending on whether there is an association or not, therapeutic interventions should be conceptualized not only targeting at behavioral auditory discrimination, but also at preattentive auditory discrimination, which has been shown to be associated with DLD (Chen et al. 2016; Cheng et al. 2021).

### 1.5 Goals, Research Questions, and Hypotheses

As discrimination of quantities is crucial for understanding the Estonian language, we aimed at investigating the maturation of duration and pitch contour perception, the two main cues for distinguishing between quantity degrees, in Estonian children with DLD compared to their TD peers across 1 year. Furthermore, we were interested in whether behavioral discrimination abilities are reflected in the MMR's amplitude in both groups. To address the aims of our study, we investigated preschool children longitudinally, with two measurements conducted 1 year apart (age at first measurement: 4;6–6;5 years; age at second measurement: 5;6–7;5 years). We studied participants' auditory processing both behaviorally as well as neurophysiologically, by measuring the MMR in the ERPs. We used a multifeature paradigm to elicit MMRs, in which the standard stimulus was a meaningful word produced in Q2. The first deviant consisted of the same segmental sequence produced in Q1 and differed from the standard in the syllable duration ratio. The second deviant consisted of the same segmental sequence produced in Q3 and differed from the standard in both the syllable duration ratio and pitch contour.

The few studies investigating duration change detection in linguistic stimuli at preschool age show that the ability to detect duration changes is still developing, as evidenced by neurophysiological measures (Linnavalli et al. 2018; Lovio et al. 2009; Pakarinen et al. 2009; Themas, Nickel, et al. 2025). This is why we expected the ability to detect changes in syllable duration to mature over the course of 1 year in both groups as indicated by a more negative MMR at T2 compared to T1 (Themas, Nickel, et al. 2025). As there is limited and contradictory evidence on the detection of the combined changes in duration and pitch contour (Lyu et al. 2024; Themas, Nickel, et al. 2025; Wu et al. 2025), we did not formulate specific hypotheses concerning the maturation of the MMR in response to duration and pitch contour changes in linguistic stimuli over the course of 1 year. Finally, it should be noted that, for both duration and combined duration‐pitch discrimination, the results may be influenced by the use of meaningful words as stimuli. In this case, the observed MMR may reflect not only changes in prosodic features but also differences in lexical meaning.

The one study investigating duration discrimination differences in participants with DLD and TD children with comparable age as in the present study used tone stimuli and reported no group differences in MMR amplitudes (Uwer et al. 2002). As several studies have shown differences in the MMRs to nonlinguistic and linguistic stimuli (A. Chen et al. 2022; Kuuluvainen et al. 2016; Paquette et al. 2015), comparability to the present study is not warranted. Literature on DD provides additional insights on the expected group differences, showing reduced negative MMR amplitudes compared to TD children (Männel et al. 2017; Schaadt and Männel 2019). This is why we expected the group differences in the MMR amplitude to be significant and that children with DLD would show a less pronounced maturation of the MMR over the course of 1 year, reflected in a less negative MMR at the second measuring time point.

Understanding the relationship between behavioral and neurophysiological measures of auditory discrimination is essential for determining whether therapeutic interventions should target not only behavioral but also preattentive auditory processing. Moreover, examining ERPs alongside behavioral measures can help validate each approach and ensure that both accurately capture auditory discrimination. To our knowledge, longitudinal investigations of this relationship are rare (e.g., Nan et al. 2018) and cross‐sectional studies provide mixed evidence on the relationship between behavioral and neurophysiological measures of auditory discrimination (Bishop et al. 2010; Halliday et al. 2014; McArthur and Bishop 2005; Pihko et al. 2006; Uwer et al. 2002). Therefore, without formulating any specific hypothesis, we investigated the association between behavioral and neurophysiological auditory discrimination exploratorily, anticipating small effect sizes given that we are working with noisy child data, potentially lacking reliability at the individual level.

## 2 Materials and Method

The current research was preregistered on the Open Science Framework, including the study description, goals, hypotheses, and a condensed version of the methods section at https://doi.org/10.17605/OSF.IO/PCW98 referenced as Themas, Schaadt, et al. (2025).^1^ All procedures performed in this study involving human participants were conducted in accordance with the ethical standards of the institutional and/or national research committee and with the 2013 revision of the World Medical Association Declaration of Helsinki, as published on the website of the Journal of the American Medical Association. The study was approved by the Ethics Committee of Human Research of the University of Tartu (Approval Number: 383/M‐12). Written informed consent was obtained from the parents or legal guardians of all participating children prior to their inclusion in the study.

This longitudinal project included a recruitment phase, a first assessment (T1), and a second assessment (T2; see Figure 1). The recruitment procedure and assessment methodology have been described in Themas, Nickel, et al. (2025), where the results of T1 are reported. As identical assessment procedures were also used for our previous work, published in Themas, Nickel, et al. (2025), we will give a concise overview of the materials, method, and procedure in the following.

**FIGURE 1:**
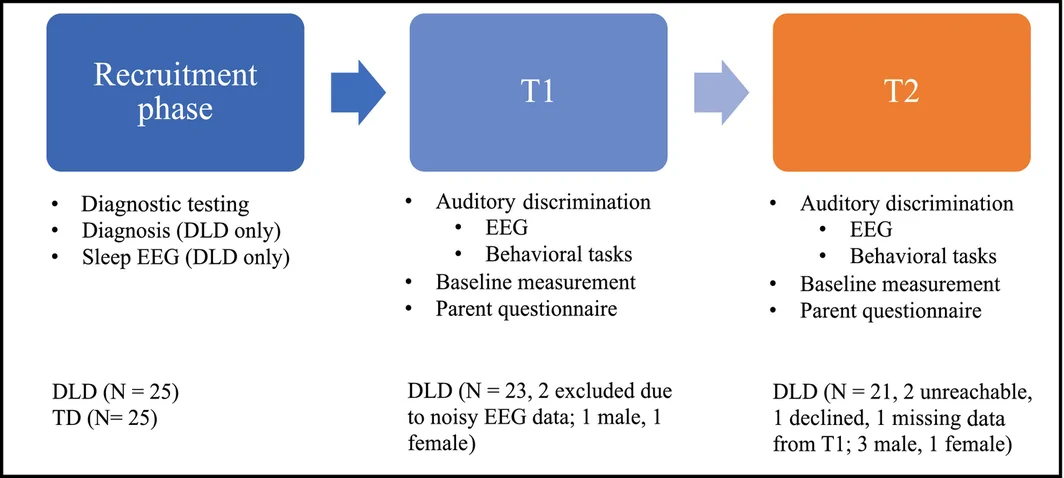
The stages of the longitudinal study. Recruitment phase, participant selection; *N*, number of participants; T1, experimental measurement at time one; T2, experimental measurement at time two.

### 2.1 Participants

The participants were Estonian‐speaking preschool children aged 4 years 6 months to 7 years 5 months. They were divided into two groups: 25 children with DLD and 25 age‐ and sex‐matched TD peers (28 males and 22 females in total). Diagnoses of DLD were established for 25 participants by an interdisciplinary clinical team consisting of a child neurologist, a clinical psychologist, and a speech and language therapist, based on the criteria of the International Classification of Diseases (10th Revision; World Health Organization 1992). The diagnostic process included language assessment, nonverbal IQ testing, and a comprehensive neurological and physical examination, including awake and sleep EEG to exclude epilepsy. Exclusion criteria for both groups included intellectual disability, sensory impairment, neurological or psychiatric diagnoses (other than language disorders), and a multilingual language environment.

Recruitment began in autumn 2021 and continued until spring 2023, during which the initial cohort was enrolled at ages 4;6–6;5 years. Participants in the DLD group were recruited through the Children's Clinic of Tartu University Hospital and from kindergartens in southern Estonia, while typically developing (TD) children were recruited from kindergartens in the same region. At the first assessment (T1), two participants were excluded due to EEG artefacts, such that the sample then comprised 48 children (see Figure 1).

The second assessment (T2) was conducted 1 year later (DLD group after M = 12.3 months, SD = 0.5; TD group after M = 12.2 months, SD = 0.7), when the children were aged 5;6–7;5 years. All participants (*N* = 50), including those excluded from T1, were invited for follow‐up. However, three participants from the DLD group did not attend the second assessment, including one who had already been excluded at T1. Thus, in total, one participant had missing data at T1, two at T2, and one at both time points. As all missing cases belonged to the DLD group, the data were assumed to be not missing at random (NMAR), and initially planned imputation methods (e.g., Multivariate Imputation by Chained Equations; Buuren and Groothuis‐Oudshoorn 2011) were therefore not applied. Instead, participants were excluded from further analyses, resulting in a final sample of 46 participants at the second assessment and an attrition rate of 8% (see Figure 1).

### 2.2 Diagnostic Testing and Background Information

To assess all children's language abilities as well as to assess DLD, tasks of the standardized language battery for 5‐ to 6‐year‐old Estonian speaking children (Padrik et al. 2013) testing phonology, vocabulary, grammar usage, pseudoword repetition, sentence repetition, and sentence comprehension were conducted. The above‐mentioned tasks were selected based on their sensitivity in differentiating atypical from age‐appropriate language development, while ensuring coverage of all major language domains.

To control for children's intellectual abilities, the Wechsler Preschool and Primary Scale of Intelligence fourth edition of the United Kingdom version (WPPSI‐IV UK; Wechsler 2013) was used for DLD and TD children at T1. As children with DLD are disadvantaged by tasks requiring language abilities, we assessed only the nonverbal intelligence and used those subtests that cover domains of visuospatial abilities, working memory, processing speed and fluid reasoning (Wechsler 2013). Importantly, assessments of intellectual abilities and language were conducted only at the initial stage of the study to characterize the sample and confirm grouping, rather than to serve as outcome measures.

A parental questionnaire was developed for this study to collect information on family background, home language environment, caregiver emotional state, attitudes toward language impairment, and attendance in speech and language therapy. The questionnaire was organized into the following five sections: (1) family background, (2) home language use, (3) caregiver well‐being, (4) attitudes toward language impairment, and (5) therapy attendance. The last two sections were included only for parents of children in the DLD group. Items were a mix of Likert‐scale ratings, multiple‐choice questions, and short open‐ended questions. The questionnaire was piloted with a small group of parents to ensure clarity. It was completed in an online format by the main caregiver at both T1 and T2.

### 2.3 Neurophysiological Experiment

#### 2.3.1 Paradigm and Stimuli

The method and stimuli used in the current study were identical to those in our previous work (Themas, Nickel, et al. 2025). Below, we provide a brief description of the paradigm and stimuli for clarity. To test for discrimination abilities of quantity degrees, a passive multifeature paradigm (Näätänen et al. 2004; Pakarinen et al. 2007; Pakarinen et al. 2009; see Figure 2A) was applied.

**FIGURE 2:**
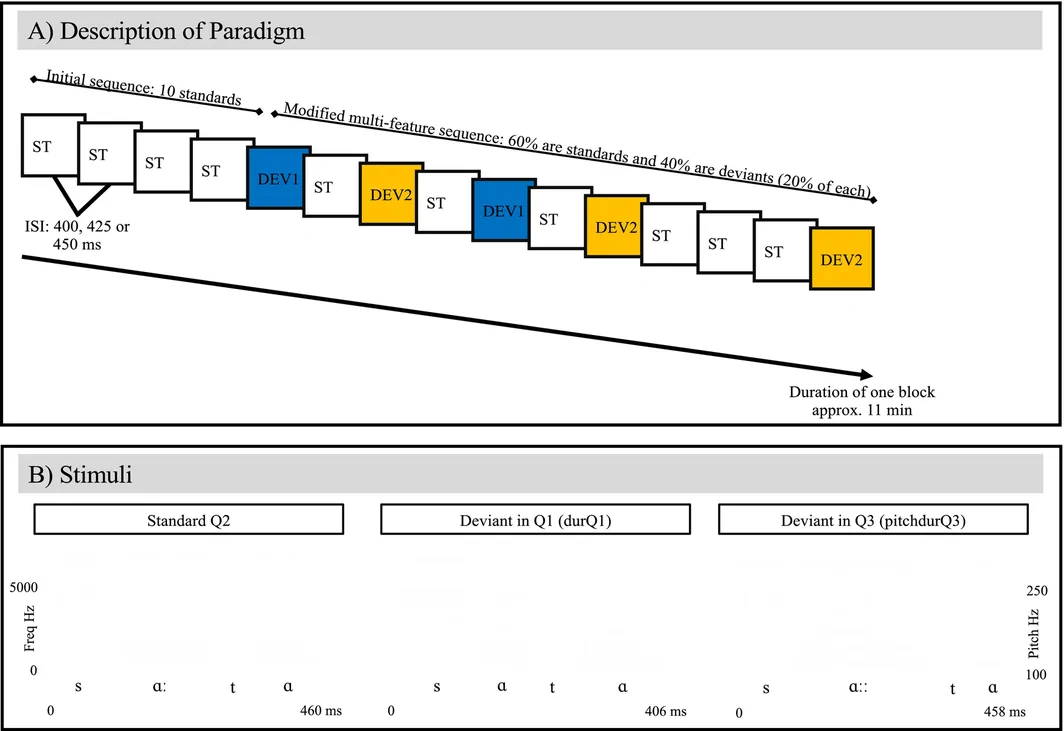
Description of stimuli and experimental procedure. (A) Structure of the stimulus presentation with ST indicating the standard stimulus and DEV indicating the decent stimulus. (B) The wave, spectrogram, pitch contour (blue line on spectrogram), and transcription of the stimuli.

The detailed description of the stimuli can be found in Table 1 and Figure 2B. We used meaningful words as stimuli, as prior research suggests that phonological features, including prosody, are more reliably discriminated in meaningful words than in pseudowords (Campos et al. 2023; Zora et al. 2015). Furthermore, high‐frequency words are processed more efficiently than low‐frequency ones (Alexandrov et al. 2011; Aleksandrov et al. 2017; Strotseva‐Feinschmidt et al. 2015). In addition, following this approach, we were able to increase ecological validity. However, potential confounds in such MMR paradigms should be considered, as changes in prosodic features (e.g., duration and pitch) inherently entail changes in lexical meaning in Estonian.

**TABLE 1:** Characteristics of the standard and two deviant stimuli.

Stimulus name	Type	Quant. degree	Total duration	1st syllable duration	Pitch fall
standard	ST	Q2	460	270	200
durQ1	DEV 1	Q1	405	190	200
pitchdurQ3	DEV 2	Q3	458	316	150

Abbreviations: 1st syllable duration, the duration of the first syllable in the stimulus in milliseconds; Quant. degree, quantity degree; Pitch fall, the approximate start of the fall of the pitch contour after stimulus onset in milliseconds; Stimuli name, names that the stimuli will be referred to; Total duration, total duration of the stimulus in milliseconds.

The standard was an Estonian word [sɑːtɑ] (*“send!, imp. sg2”*), which is pronounced in the second quantity degree (Q2). For the first deviant (henceforth durQ1), we changed the quantity degree of the standard to be in Q1 [sɑtɑ] (syllable duration ratio change), which also resulted in the change in meaning (*“hundred, nom. sg”*). For the second deviant (henceforth pitchdurQ3), we changed the quantity degree of the standard to be in Q3 [sɑːːtɑ] (both syllable duration ratio and pitch contour change), which also resulted in the change in meaning (*“to get, inf.”*).

The stimuli were naturally spoken by a female native Estonian speaker (the first author of the paper) in a soundproof room. Target words were segmented from carrier phrases using PRAAT (Version 6.1; Boersma and Weernink 2021). These words were embedded in the middle of the phrase (e.g., *ütle .. kõvasti “*say … loudly*”* and *“*say a hundred loudly*”*) to maintain natural intonation patterns.

In the context of a larger project, participants were presented with three different stimulus blocks (total duration 35 min), and the order of the blocks was changed for each participant. Therefore, we did not expect any systematic influence of the order of the blocks. Here, we report the results from one of these blocks (duration 11 min), in which a total of 1100 stimuli with alternating interstimulus interval (400, 425, or 450 ms; Figure 2A) were delivered through headphones using MATLAB (The MathWorks 2015; Version 8.5.0). We used the multifeature paradigm with 60% of standards and 40% of deviants in which the stimuli were presented such that the number of standards between the deviants was either one or three (see Figure 2A) to investigate habituation differences between children with DLD and their TD peers (results presented elsewhere). The multifeature paradigm has been successfully used in several studies with children of different ages and with different clinical populations (Kuuluvainen et al. 2016; Linnavalli et al. 2018; Niemitalo‐Haapola et al. 2015; Partanen, Torppa, et al. 2013; Partanen, Pakarinen, et al. 2013; Schaadt et al. 2023; Torppa et al. 2022; Virtala et al. 2022), showing similar MMN/MMR amplitudes compared to a typical oddball paradigm (Lovio et al. 2009; Pakarinen et al. 2009; Partanen, Pakarinen, et al. 2013). Importantly, we used less deviants (see also Fisher et al. 2011; Thiede et al. 2019; Virtala et al. 2022) compared to the typically used five deviants to reduce exhaustion of the auditory system, which might be particularly the case for clinical populations (Kujala et al. 2006; Partanen, Pakarinen, et al. 2013).

#### 2.3.2 EEG Recording

The EEG was recorded from 32 active electrodes (ActiveTwo system, BioSemi B.V., Amsterdam, Netherlands) placed at standard positions according to the 10–20 system, with the signal referenced to the CMS electrode and common mode rejection handled by the DRL electrode during data acquisition. Electrooculograms were recorded from two electrodes at the outer canthi of both eyes, one electrode on the orbital ridge under the right eye near the lower eye socket and one electrode located near the supraorbital ridge above the right eye, slightly lateral to the midline of the forehead. The EEG data were digitized at 512‐Hz frequency with a bandpass filter of 0.16–100 Hz.

### 2.4 Behavioral Tasks

#### 2.4.1 Quantity Discrimination Task

The computerized quantity discrimination task included three sets of naturally produced, meaningful Estonian disyllabic words, each representing all three quantity degrees (set one—Q1 [vɑlɑ] “*pour!* imp. 2Sg”, Q2 [vɑ:lɑ] “*whale*, gen. Sg”, Q3 [vɑ::lɑ] “*whale*, part. Sg”; set two—Q1 [ketɑ] “*who*, part. Sg”, Q2 [ke:tɑ] “*boil*, imp. 2Sg”, Q3 [ke::tɑ] “*to boil*, infinitive”; set three—Q1 [pole] “*is not*”, Q2 [po:le] “*half*, gen. Sg”, Q3 [po::le] “toward”). An extra six stimuli were chosen for the training phase. The task was to actively listen and press a button as soon as a new stimulus was heard in the sequence of stimuli. The stimuli were presented in the context of a roving standard paradigm (Fong et al. 2020), where a stimulus was repeated 3–5 times to establish it as the perceptual baseline, followed by the introduction of a new stimulus with a different quantity degree. This new stimulus was again repeated 3–5 times until it became the new baseline. The three sets of stimuli were presented in separate blocks, and the order of the blocks was counterbalanced across participants. The interstimulus interval was 2000 ms, and participants heard approximately 100 stimuli during the four‐and‐a‐half‐minute session. To ensure task comprehension, testing was preceded by a training phase during which the researcher guided the participant through the same procedure using different stimuli.

#### 2.4.2 Lexical Decision Task

The computerized lexical decision task included 15 nouns and 15 pseudowords. The nouns were high‐frequency Estonian words considered to be familiar to 4‐year‐olds with a CV(::)CVC structure. Each of the three quantity degrees were evenly represented in the used nouns and pseudowords. The pseudowords were created by changing the quantity degree of nouns (e.g., word—Q2 [rɑ:mɑt:] *a book*; pseudoword—Q3 [rɑ::mɑt:]), yielding five pseudowords per degree (see stimuli in Appendix A). Participants responded by pressing a button when the spoken word was meaningful and withheld their response when the stimulus was a pseudoword. All nouns and pseudowords were presented once in a randomized order, with the constraint that corresponding word‐pseudoword pairs were not presented consecutively. To support children's performance, each auditory stimulus was paired with a visual image. The interstimulus interval was 3000 ms and the testing lasted approximately 3 min. To ensure task comprehension, testing was preceded by a training phase during which the researcher guided the participant through the same procedure using different stimuli.

It should be noted that the present task does not directly assess discrimination, as it also involves higher‐level cognitive processes, such as accessing lexical representations from the mental lexicon. This task was chosen because it allowed for a less abstract and more child‐friendly design through the use of visual stimuli. Additionally, the task instruction itself (i.e., judging whether the word is correct or not) is relatively easy for children to understand, as similar paradigms used in studies of mispronunciation detection have been shown to be accessible even for 4‐year‐old children (Rvachew and Brosseau‐Lapré 2018). In contrast, the quantity discrimination task is more abstract and less engaging and may therefore be too demanding for younger children. Therefore, the lexical decision task was included as an additional measure, designed to provide an alternative means of assessing auditory discrimination if needed.

### 2.5 Procedure

The study started with the recruitment phase during which the children with DLD received diagnostic testing at the Children's Clinic of Tartu University Hospital. The diagnostic testing of the TD group was carried out in the experimental psychology laboratory of Tartu University by the first author and a psychologist with at least a master's level of education. Testing was conducted over two sessions, each lasting slightly more than 1 h, for a total duration of approximately 2.5 h.

Approximately 2 weeks after recruitment, T1 was conducted at the Laboratory of Experimental Psychology at the University of Tartu, consisting of a neurophysiological experiment and behavioral tasks. During the neurophysiological session, participants were seated comfortably in front of a monitor. They watched a silent cartoon of their choice during the EEG cap fitting and experimental tasks. The stimuli were presented using a comfortable volume level between 55 and 65 dB with JBL Tune290 earphones. After the neurophysiological experiment, the cap was removed, and the participants proceeded to the behavioral tasks. They were seated in front of a computer next to the experimenter, who helped the children through the teaching phases of the behavioral tasks and remained with the child during the testing phases. The stimuli were presented using a comfortable volume level between 55 and 65 dB with JBL Tune290 earphones. Throughout the whole procedure, a parent was always present in the laboratory. The total duration of the assessment was approximately 1 h and 15 min.

### 2.6 Data Analysis

The raw EEG data that support the findings of this study are openly available in the University of Tartu Library DataDOI repository at https://doi.org/10.23673/re‐525, referenced as Themas (2025). The code and data for modelling are available in the Zenodo repository at https://doi.org/10.5281/zenodo.15799644, referenced as Themas and Nickel (2025).

#### 2.6.1 Descriptive Statistics of the Sample

The Wilcoxon rank‐sum test was used to compare the two participant groups with respect to age, standardized language test scores, and WPPSI‐IV UK nonverbal IQ scores. For variables like sex, handedness and the level of maternal education (basic education—completed 9 years of school; secondary education—completed 12 years of school; vocational education—providing specialized skills and knowledge for a particular trade or occupation; higher education—at least a bachelor's degree), we used the Chi‐squared test.

#### 2.6.2 EEG Data Processing and Analysis

The Maryland analysis of developmental EEG (MADE; Debnath et al. 2020) was used to preprocess EEG data in MATLAB (MathWorks 2023; Version 9.14) using EEGLAB (Version 2023.1; Delorme and Makeig 2004). The data were recorded with a sampling rate of 512 Hz and down sampled to 500 Hz following high (0.3 Hz, stopband of 0.1 Hz) and low (50 Hz, transition band of 10 Hz) pass filtering. Global artefact rejection was performed using the FASTER extension in EEGLAB (Nolan et al. 2010). Any participant whose dataset contained more than 10% of bad channels was removed from the study. Muscle and eye‐movement artefacts were rejected using independent component analysis (ICA) and the components, which corresponded to artefacts were selected using the adjusted‐ADJUST algorithm (Leach et al. 2020). The data were segmented into epochs time‐locked to stimulus onset. The chosen epoch window (−100 to 700 ms) was shorter than the stimulus onset asynchrony (~910 ms), ensuring that each epoch captured responses to a single stimulus without overlap from subsequent events. The epochs were then baseline corrected using the 100‐ms prestimulus interval. Residual artefacts were identified by scanning electrodes for epochs in which voltage values surpassed ±125 μV. The missing channels were interpolated. Lastly, the data were re‐referenced using an average reference.

Consistent with T1 (Themas, Nickel, et al. 2025), no significant group differences were found in the number of usable epochs (see Table 2) at the second assessment. Grand averages of the ERPs to the standard and deviant stimuli as well as the difference waveform were plotted using EEGLAB (Version 2023.1; Delorme and Makeig 2004; see Figures 3 and 4).

**TABLE 2:** Means, minimum values, maximum values, and standard deviation of the usable epochs by group and sex at T1 and T2.

	Mean		Min		Max		SD	
	T1	T2	T1	T2	T1	T2	T1	T2
DLD	624	577	456	125	734	718	76	149
TD	627	599	417	273	706	729	71	133
Male	623	613	417	349	734	729	73	118
Female	617	555	456	125	704	725	72	163

**FIGURE 3:**
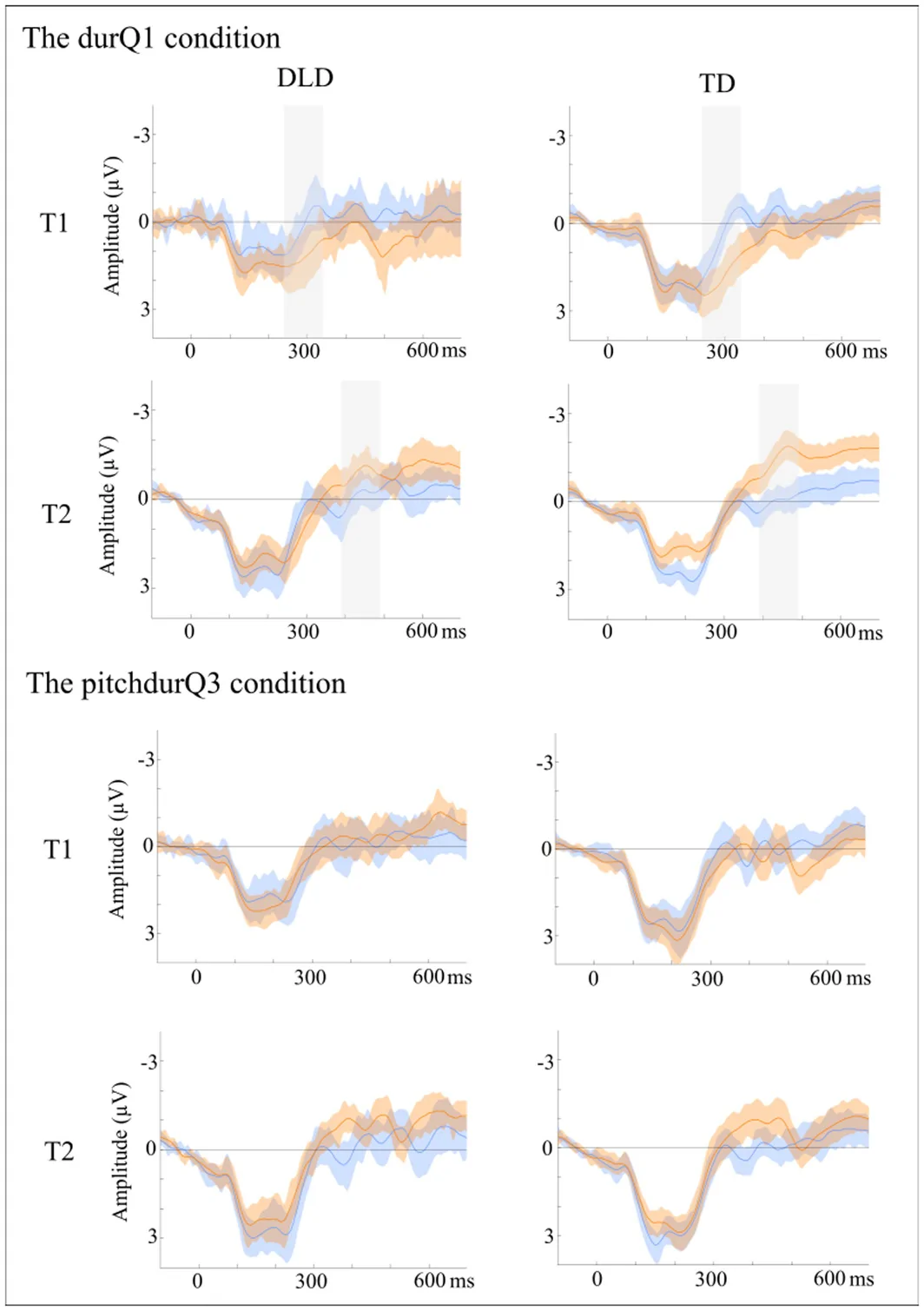
ERPs to the standard (blue) and deviant (orange) stimuli at T1 and T2 separately for children with and without DLD. Lighter shaded areas surrounding the waveforms represent the corresponding 95% confidence intervals. DurQ1 condition—depicted are the mean grand average waveforms and their corresponding confidence intervals across all electrodes showing significant effects in the cluster‐based permutation test. The gray‐shaded areas indicate time windows with statistically significant differences between ERPs to the standard and deviant stimuli. PitchdurQ3 condition—depicted are the mean grand average waveforms and their corresponding confidence intervals across electrodes commonly employed in MMR analyses (F3, F4, FC, C3, C4, and Cz), as no significant deviant‐standard difference was found in the cluster‐based permutation testing. For visualization purposes, the waveforms were smoothed using a 15‐point moving average (equivalent to 30 ms at a 500‐Hz sampling rate).

**FIGURE 4:**
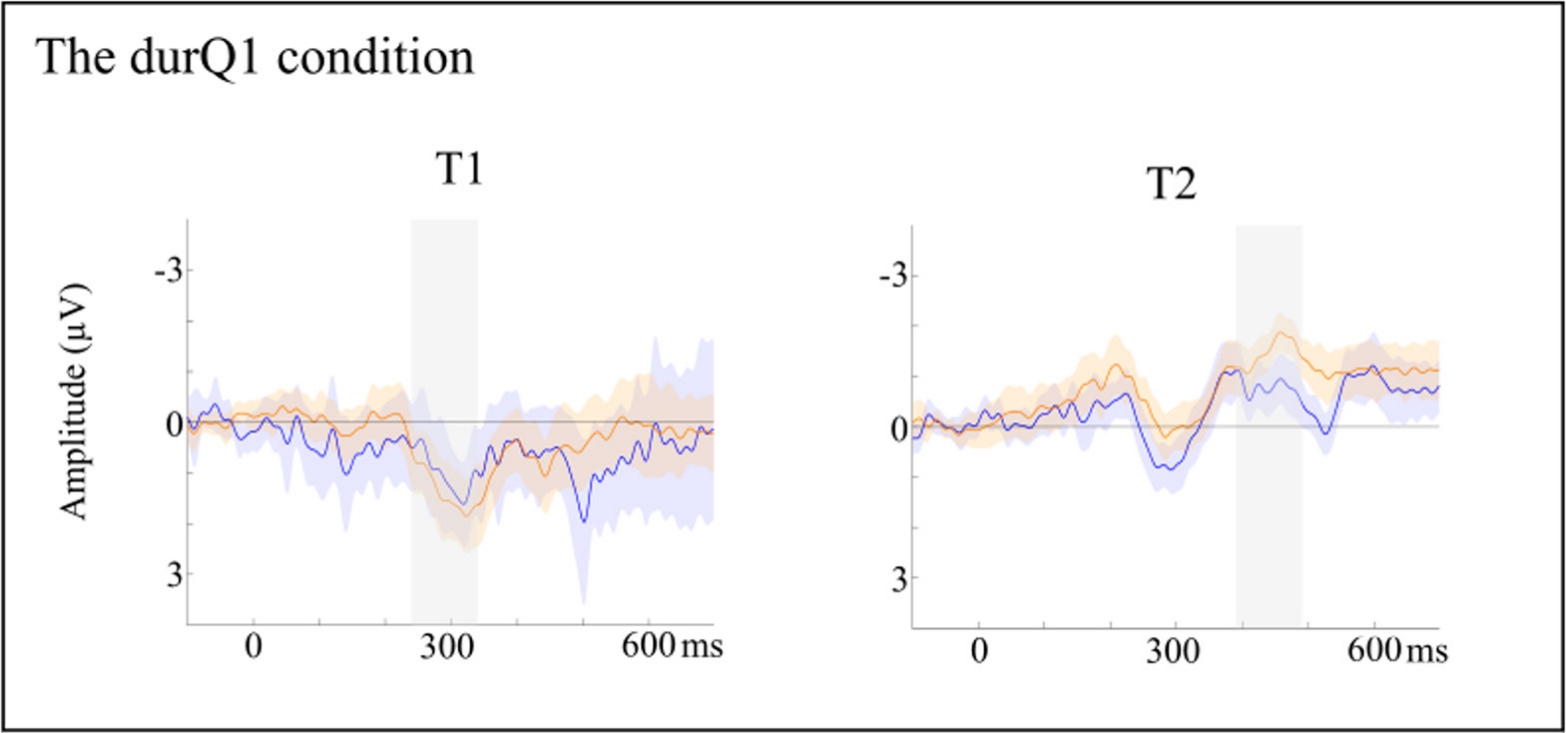
Difference waves (deviant minus standard) at T1 and T2 separately for children with and without DLD. Depicted are the grand averages of the difference waves across all electrodes showing significant effects in the cluster‐based permutation test. The gray‐shaded areas indicate the time window of statistically significant differences between ERPs to the standard and deviant stimuli in the *durQ1* condition.

To identify time windows and electrodes where ERPs to the standard and deviant stimuli significantly differed, that is the MMR, cluster‐based permutation tests (MATLAB, Fieldtrip toolbox; Oostenveld et al. 2011) were calculated for each time point between 90 and 698 ms within epochs. Only the largest positive and negative clusters representing the deviant‐standard difference were considered and quantified for further analysis, as this approach controls for the family‐wise error rate and minimizes the likelihood of false discoveries. The method and the code are based on Meyer et al. (2021).

We used linear mixed models (LMMs) to analyze longitudinal changes in MMR amplitude, group differences of maturational changes, and the relationship between behavioral and neurophysiological measures. The statistical modelling strategy followed Murphy et al. (2022) and was implemented in R (Version 4.3.2; R Core Team 2023) using the *lme4* package (Version 1.1–35.3; Bates et al. 2015) and *lmerTest* (Version 3.1–3; Kuznetsova et al. 2017), with visualizations created using *ggplot2* (Version 3.4.4; Wickham 2016). In all of the LMMs, the MMR difference wave amplitudes (deviant—standard) served as a dependent variable. A random intercept was included to account for the correlation between repeated measures, and a random slope for time was added to capture anticipated variations in slopes over time among participants.

In order to capture potential group differences, two separate LMMs were fitted analyzing changes in MMR amplitude over the course of 1 year across all children. Separate LMMs were fitted for each deviant condition (durQ1 and pitchdurQ3), each including a three‐way interaction between time window (significant clusters as described above), time (T1 vs. T2), and group (DLD vs. TD). Additionally, participant age at T1 was also tested as a continuous independent variable, but the more complex model including participant age and the three‐way interaction did not provide a better fit and was therefore not retained in the final model. Post hoc analyses using estimated marginal means (*emmeans*; Lenth 2024) were performed after fitting the LMM to probe the interaction effects observed in the model, as the model output alone does not directly provide the specific group, time window, and time‐point––based comparisons of interest. Finally, the marginal and conditional pseudo R‐squared (*R* ^2^) values were examined to improve the interpretability of the models.

The models were fitted with maximum likelihood estimation and degrees of freedom were approximated using the Satterthwaite method. For model diagnostics, we checked for potential multicollinearity, nonlinearity between dependent and independent variables, heteroscedasticity in the model's residual variance and normal distribution of the model's residuals, random effects, and random intercepts.

#### 2.6.3 Behavioral Data and Behavioral–Neurophysiological Relationship Analyses

For each behavioral task, we calculated the sensitivity index (*d*′) for each participant. This index reflects performance accuracy, as shown in the equation below. *Z* (hit rate) represents how many standard deviations the observed hit rate was from the mean of the standard normal distribution and the *Z* (false alarm rate) indicates how many standard deviations the observed false alarm rate was from the mean of the standard normal distribution.

*d*′ *= Z* (Hit Rate) − *Z* (False Alarm Rate).

To examine changes in performance over time, within‐participant differences in *d*′ scores between T1 and T2 were assessed using Wilcoxon signed‐rank tests. Between‐group differences in overall performance (*d*′ scores) between DLD and TD participants were evaluated using Wilcoxon rank‐sum tests.

To further characterize participants' sensitivity to different types of quantity contrasts, performance was analyzed separately for each contrast pair: first quantity degree (Q1) versus second quantity degree (Q2; syllable duration difference), first quantity degree (Q1) versus third quantity degree (Q3; large syllable duration difference and pitch contour difference), and second quantity degree (Q2) versus third quantity degree (Q3; small syllable duration difference and pitch contour difference). For these analyses, errors in contrast discrimination were counted and summed across participants. In the quantity discrimination task, an error was defined as the absence of a button press in response to a change in quantity. In the lexical decision task, an error was defined as a button press in response to a word that was incorrectly accepted despite being produced with an incorrect quantity degree. Finally, group differences were assessed using Wilcoxon rank‐sum tests.

To examine the association between behavioral task results and MMR amplitude, we created separate models in which the predictors were: the *d*′ scores from the quantity discrimination task at T1 and T2 and the *d*′ scores of the lexical decision task at T1 and T2.

Two different model structures were tested, with each of the four *d*′ scores. In the first model (MMR ~ time window × time × *d*′), it was examined whether there was an overall relationship between the MMRs and the *d*′ scores. In the second model (MMR ~ time window × time + *d*′ × group), it was examined whether the relationship between MMRs and *d*′ scores differed between groups. Similar to the previous LMMs, the current ones were fitted with maximum likelihood estimation, degrees of freedom were approximated using the Satterthwaite method, and the same model diagnostics was applied.

## 3 Results

### 3.1 Sample Statistics

The description of age, sex, handedness, maternal education level, language test scores and nonverbal IQ scores for the two groups are presented in Table 3. After excluding four participants from the original DLD sample (Themas, Nickel, et al. 2025), children with DLD did not differ significantly from their TD peers in age, sex, or handedness. As in the T1 sample, they did differ in maternal education level, standardized language test scores, and WPPSI‐IV UK nonverbal IQ scores (see Table 3).

**TABLE 3:** Sample statistics.

	DLD	TD	Group diff.
T1	*n* = 23	*n* = 25	
	Mode	Mode	(χ^2^, df) *p*
Sex	Male (*n* = 13)	Male (*n* = 14)	(0, 1) 1
Mat. edu.	Sec. (*n* = 8)	High. (*n* = 22)	(18, 3) 0.00036
Hand.	Right (*n* = 22)	Right (*n* = 22)	(7.2 × 10^−32^, 1) 1

	Mean	SD	Mean	SD	(*W*) *p*
Age	69	6.0	69	6.2	(306) 0.92
Lang. test	20	6.9	36	4.7	(535) 3.0 × 10^−8^
Nonverbal IQ	96	8.3	108	13	(421) 0.0019

T2	*n* = 21	*n* = 25	
	Mode	Mode	(χ^2^, df) *p*
Sex	Male (*n* = 12)	Male (*n* = 14)	(0, 1) 1
Mat. edu.	Sec. (*n* = 7); High. (*n* = 7)	High. (*n* = 22)	(15, 3) 0.0016
Hand.	Right (*n* = 20)	Right (*n* = 22)	(0.12, 1) 0.73

	Mean	SD	Mean	SD	(*W*) *p*
Age	82	6.0	81	5.8	(283) 0.67

*Note:* Sex, maternal education, and handedness are reported as the most frequent category (mode) within each group. Age is reported in months. Lang. test at T1, scaled overall score of the language test. Nonverbal IQ at T1 = overall scaled score of the WPPSI‐IV UK nonverbal IQ measure.

Abbreviations: Hand., handedness; Mat. edu., maternal education level: sec., secondary education.

### 3.2 Results of the EEG Data Analysis

Grand averages of the ERPs to the standard and deviant stimuli (durQ1) as well as of the difference waveform (deviant durQ1—standard) at T1 and T2 separately for children with and without DLD can be found in Figure 3 and Figure 4. The cluster‐based permutation tests (see plots in Appendix B) done at T1 revealed a more positive amplitude in response to the deviant stimulus compared to the standard stimulus between 240 and 340 ms after stimulus onset (i.e., 140–240 ms after deviance onset) at frontal, fronto‐central and central electrodes (AF3, F3, FC5, F7, Fz, FC1, C3, FC6, and F8). The mean MMR values (deviant‐standard) and their standard deviations for this significant cluster are presented in Table 4.

**TABLE 4:** Means and standard deviations of the amplitudes of the MMRs at T1 and T2.

	Correlation T1 and T2	DLD				TD			
	(*r*, df) *p*	Mean		SD		Mean		SD	
		T1	T2	T1	T2	T1	T2	T1	T2
Q1	(−0.10, 44) 0.50	1.01	−0.83	1.94	0.96	1.34	−1.4	1.56	0.86
Q3	(−0.0073, 44) 0.96	−0.56	−1.4	2.28	2.1	−0.18	−1.0	1.48	1.7

*Note:* The values represent difference waveforms (deviant minus standard) in microvolts (μV). Pearson's product–moment correlation was calculated between the mean MMR amplitudes at T1 and T2.

Abbreviations: Q1, MMR in the duration change condition (durQ1); Q3, MMR in the duration and fundamental frequency contour change condition (pitchdurQ3).

At T2 the cluster‐based permutation tests (see plots in Appendix B) revealed a more negative amplitude in response to the deviant stimulus compared to the standard stimulus between 390 and 490 ms after stimulus onset (i.e., 290–390 ms after deviance onset) at frontal, fronto‐central, central, centro‐parietal and parietal electrodes (F3, F4, FC1, FC2, C4, Cz, CP2, and Pz). Both the above‐mentioned time‐windows and electrodes were further analyzed with LMMs. For further significant clusters, not related to the MMR, see Appendix C. The mean MMR values (deviant‐standard) and their standard deviations for this significant cluster are presented in Table 4.

Grand averages of the ERPs to the standard and deviant stimuli (pitchdurQ3) at T1 and T2 separately for children with and without DLD can be found in Figure 3. The cluster‐based permutation tests (see plots in Appendix B) revealed no time windows or electrodes where the waveforms of the standard and deviant stimuli were significantly different at T1, which was also the case after 1 year in the current study. Therefore, we did not further analyze the pitchdurQ3 condition in the present study.

To enable robust modelling of within‐participant variation and the inclusion of participant as a random intercept, multiple mean MMR values were extracted per participant. For each measurement (T1 and T2), two MMR values were extracted from the positive cluster (means from 140‐ to 190‐ and 190‐ to 240‐ms time windows) and two from the negative cluster (means from 290‐ to 340 and 340‐ to 390‐ms time windows). Accordingly, the LMM included eight mean MMR values per participant, yielding a total of 369 observations.

The results of the LMM are reported in Table 5. The fixed effects alone accounted for approximately 26% of the variance (marginal *R* ^2^ = 0.26), while the full model, including both fixed and random effects, explained approximately 54% (conditional *R* ^2^ = 0.54).

**TABLE 5:** The results of the MMR linear mixed model of the duration deviant condition.

	Estimate	Standard error	df	*t* value	95% CI	*p*
Intercept	0.6	0.29	68	2.06	0.03; 1.16	0.044*
TW2	−0.85	0.25	276	−3.48	−1.33; −0.37	5.93 × 10^−4^***
Time2	−1.22	0.33	84	−0.37	−0.78; 0.53	0.71
GroupTD	0.75	0.39	68	1.91	−0.02; 1.52	0.061
TW2: Time2	−0.32	0.35	276	−0.92	−0.99; 0.36	0.36
TW2: GroupTD	−0.87	0.33	276	−2.62	−1.52; −0.22	9.28 × 10^−3^**
Time2: GroupTD	−0.56	0.45	84	−1.24	−1.44; 0.33	0.22
TW2: Time2: GroupTD	0.06	0.47	276	0.12	−0.87; 0.98	0.91

*Note:* The intercept is TW1, Time 1, and Group DLD.

Abbreviations: TW, time window 1 (positive cluster) and 2 (negative cluster); Time, 1 and 2 (measurements 1 and 2); Group, DLD and TD.

Significance codes: ***p < 0.001, **p < 0.01, *p < 0.05, . *p* < 0.1, *p* ≥ 0.1.

Post hoc analyses indicated distinct longitudinal patterns across clusters. When data were collapsed across groups, MMR amplitude did not exhibit a reliable change over time in the positive cluster (T2‐T1: *estimate* = −0.40, SE = 0.23, *t*(88.2) = −1.75, 95% CI [−0.86, 0.06], *p* = 0.084). In contrast, the amplitude in the negative cluster became significantly more negative across the two measurements (T2‐T1: *estimate* = −0.69, SE = 0.23, *t*(88.2) = −3.01, 95% CI [−1.15, −0.23], *p* = 0.0034).

Group‐specific contrasts further revealed differential effects across measurement points. At the first measurement, MMR amplitude differed between the DLD and TD groups at a trend level in the positive cluster (TD‐DLD: *estimate* = 0.75, SE = 0.40, *t*(70.8) = 1.87, 95% CI [−1.55, 0.050], *p* = 0.066), whereas at the second measurement a significant group difference emerged in the negative cluster (TD‐DLD: *estimate* = −0.63, SE = 0.30, *t*(97.5) = −2.06, 95% CI [0.024, 1.23], *p* = 0.042).

Examination of within‐group longitudinal contrasts (see Figure 5) showed that MMR amplitudes in the DLD group remained stable over time in both the positive cluster (T2‐T1: *estimate* = −0.12, SE = 0.34, *t*(88.2) = −0.36, *p* = 0.718) and the negative cluster (T2‐T1: *estimate* = −0.44, SE = 0.34, *t*(88.2) = −1.30, *p* = 0.198), as well as when averaged across clusters (T2‐T1: *estimate* = −0.28, SE = 0.29, *t*(48.1) = −0.97, 95% CI [−0.87, 0.03], *p* = 0.339). The TD group demonstrated significant longitudinal modulation of MMR amplitude, with increasing negativity in both the positive (T2‐T1: *estimate* = −0.68, SE = 0.31, *t*(88.2) = −2.19, *p* = 0.031) and negative clusters (T2‐T1: *estimate* = −0.94, SE = 0.31, *t*(88.2) = −3.04, *p* = 0.0032), as well as in the cluster‐averaged measure (T2‐T1: *estimate* = −0.81, SE = 0.27, t(48.1) = −3.05, 95% CI [−1.35, −0.28], *p* = 0.004), where the MMR amplitude became more negative.

**FIGURE 5:**
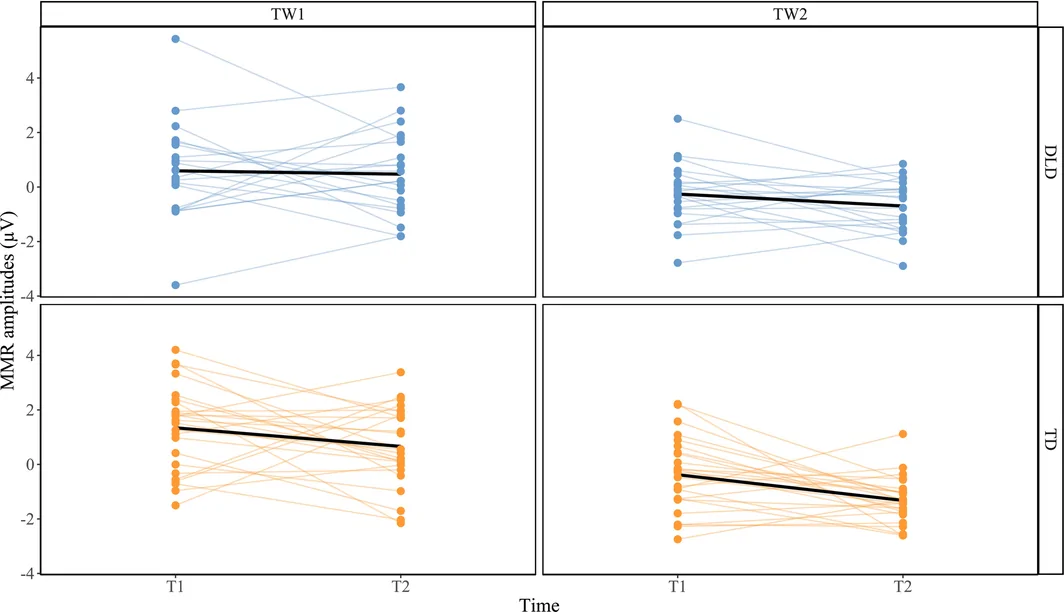
Changes in individual MMR values from T1 to T2 with mean estimates from the LMM. TW1—changes in the positive time‐window; TW2, changes in the negative time‐window; dots, individual raw MMR amplitude values at T1 and at T2; thin lines, connect each participant's mean amplitude at T1 to their corresponding value at T2; thick line, connects the mean LMM estimate of the MMR value at T1 to the corresponding estimate at T2.

Despite these within‐group differences, direct comparisons of longitudinal slopes did not provide evidence for statistically reliable group differences in the magnitude of MMR amplitude change over time, either in the positive cluster (*estimate* = −0.56, SE = 0.46, *t*(88.2) = −1.21, 95% CI [−1.47, 0.36], *p* = 0.228) or in the negative cluster (*estimate* = −0.50, SE = 0.46, *t*(88.2) = −1.10, 95% CI [−1.42, 0.41], *p* = 0.276).

### 3.3 Results of the Behavioral Data and Behavioral‐Neurophysiological Relationship Analysis

In both of the behavioral discrimination tasks, participants performed significantly better at T2 compared to T1, with higher *d*′ scores in both the quantity discrimination (*V* = 150, *p* = 3.15 × 10^−5^) and lexical decision tasks (*V* = 242, *p* = 0.0012; see Table 6), and shorter reaction times in both tasks (quantity discrimination: *V* = 650, *p* = 5.21 × 10^−5^; lexical decision: *V* = 762, *p* = 0.0011). Group means differed significantly in *d*′ scores of the quantity discrimination task and lexical decision task, but no differences in means were found in RTs (see Table 6). We further examined the discrimination of different quantity degrees. Table 6 shows the sum of errors made while discriminating specific quantity contrasts and group differences in means.

**TABLE 6:** Means and group differences in behavioral discrimination tasks.

Quant. diff.	DLD		TD		Group diff.	
	T1	T2	T1	T2	T1 (*W*) *p*	T2 (*W*) *p*
*d*′	1.7	2.3	2.7	3.4	(115) 0.0010	(105) < 0.001
RT	1718	1326	1555	1229	(190) 0.79	(319) 0.22
Q1 vs. Q2	97	43	45	22	(433) < 0.001	(360) 0.027
Q1 vs. Q3	39	32	17	4	(345) 0.054	(455) < 0.001
Q2 vs. Q3	101	50	76	36	(363) 0.027	(336) 0.10

Lex. des.	T1	T2	T1	T2	T1 (*W*) *p*	T2 (*W*) *p*
*d*′	1.6	2.3	2.6	3.0	(139) 0.0068	(119) 0.0015
RT	1731	1577	1590	1492	(329) 0.15	(293) 0.51
Q1 vs. Q2	42	21	10	8	(414) < 0.001	(366) 0.010
Q1 vs. Q3	10	6	2	3	(332) 0.028	(295) 0.218
Q2 vs. Q3	49	41	26	16	(370) 0.016	(380) 0.007

Abbreviations: *d*′, the overall discrimination performance indexed by *d*′ scores; Group diff., group differences; Lex. des., lexical decision task; Q1 vs. Q2 (and other quantity contrasts), summed errors in discriminating specific quantity contrasts; Quant. diff., quantity discrimination task; RT, reaction time; *W*, Wilcoxon rank‐sum test statistic.

No significant relationship was found between MMR amplitude and any behavioral *d*′ scores. Similarly, no group differences were observed in the relationship between MMR amplitudes and *d*′ scores.

## 4 Discussion

The aim of the present study was to investigate the maturation of duration and pitch contour perception, the two main cues for distinguishing between quantity degrees, in Estonian children with DLD compared to their TD peers longitudinally across 1 year. Furthermore, we were interested in whether behavioral discrimination abilities are reflected in the MMR amplitudes in both groups. For duration discrimination, we found an early positive MMR, which decreased over time, whereas a subsequent negative MMR increased in amplitude over time. Although these observed longitudinal changes were significant in the TD children, no such changes were evident in the DLD group. However, direct comparisons of maturational trajectories did not reveal significant group differences. In contrast, we did not find any significant MMR in response to the duration and pitch contrast in neither group and at neither assessment. Additionally, no association was found between changes in the MMR and behavioral discrimination performance at the individual level neither at first nor second assessment.

### 4.1 Maturation of the MMR

For duration discrimination (durQ1), the MMR amplitude was hypothesized to become more negative over 1 year from first to second measurement in both groups, reflecting a maturational shift (Linnavalli et al. 2018; Lovio et al. 2009; Pakarinen et al. 2009; Themas, Nickel, et al. 2025). Partially in line with this assumption, we found a positive MMR at T1 between 140 and 240 ms after stimulus onset and a negative MMR at T2, which was, however, observed within a later time window (290–390 ms) compared to the positive cluster at T1. Interestingly, the positive cluster between 140 and 240 ms had diminished at T2, which is consistent with previous studies reporting positive MMRs in 4‐ to 7‐year‐old children in a comparable time window to decrease with maturation (Lee et al. 2012; Maurer et al. 2003; Putkinen et al. 2019; Shafer et al. 2010). Further studies on the maturation of MMR in children of a similar age range to the present sample reported the emergence of negative MMRs in later time windows following an earlier positive response, sometimes occurring subsequently (Partanen, Torppa, et al. 2013; Shafer et al. 2010), which aligns with the present results. The decline of the positive MMR and the emergence of a negative MMR may indicate a polarity change of the MMR (Lee et al. 2012; Putkinen et al. 2019). Maurer et al. (2003) provided evidence that the positive MMR observed in children and the negative MMN in adults originate from similar generator regions in the temporal cortex, despite exhibiting opposite scalp polarities. That we found an increase in latency (140–240 ms at T1 and 290–390 ms at T2) might contrast with the assumption of similar generator regions (Maurer et al. 2003) and the idea of polarity change of the same component. Additionally, other studies do not report an increase in latency with maturation at preschool and early school ages (Shafer et al. 2000; Shafer et al. 2010; Themas et al. 2023). These findings suggest alternative interpretations of the positive and negative MMR, incorporating latency differences and possibly supporting the view that they represent two distinct components. Accordingly, the negative response with longer latency might be related to more sophisticated and at the same time more time‐consuming processing of the acoustic stimulus features in older children (Alatorre‐Cruz et al. 2023; Čeponienė et al. 2005; Siegler 2004). This claim is supported by studies, where both positive and negative MMR co‐exist in 3‐ to 12‐year‐olds (Lee et al. 2012; Liu et al. 2014; Partanen, Torppa, et al. 2013; Shafer et al. 2010). In line, it has been suggested that the different MMR polarities reflect distinct underlying neural processes (Rivera‐Gaxiola et al. 2005), potentially linked to bottom‐up (positive MMR) and top‐down (negative MMR) processing pathways, which are thought to involve separate neural generators (see Schaadt et al. 2015). Thus, the negative MMR at T2 may reflect more sophisticated top‐down processes that are, however, less mature and more time‐consuming in comparison to adults. To prove this assumption, an additional measurement would be needed to show that processes become more efficient, reflected in shorter MMR latencies, when children get older.

An alternative explanation for the observed latency differences could be that the positive MMR observed at T1 was not detectable at T2 due to it crossing zero caused by an ongoing polarity shift (discussed in Marklund et al. 2019). Thus, instead the negative MMR at T2 may represent a late discriminative negativity that decreased in latency from T1 (390–590 ms after deviance onset, see Appendices B and C) to T2 (290–390 after deviance onset, see Appendices B and C). The findings in the literature on the latency decrease of LDN are contradictory so far. Some report reduction of latency with age (Bishop et al. 2011; Mueller et al. 2008), some report no latency change in school aged children (David et al. 2020; Hommet et al. 2009) and some report increase in latency with age in young children (Virtala et al. 2022). We argue that our findings align with David et al. (2020) and Hommet et al. (2009), because we also observed a small but significant deviant‐standard difference cluster at T2, occurring at the same time‐window (490–590 ms after deviance onset) and over similar region as the LDN at T1 (Appendix B and C). Thus, the negativity observed between 290 and 390 ms at T2 is unlikely to reflect an LDN, but rather an MMR. Instead, the LDN at T2 appeared later and in a similar time window as at T1 and was smaller in amplitude compared to T1, possibly due to the participants being older at T2 (Bishop et al. 2011; Gumenyuk et al. 2004; Hommet et al. 2009; Linnavalli et al. 2018; Liu et al. 2014; Morales et al. 2022; Torppa et al. 2022).

Interestingly, LMM predictions in our previous study (Themas, Nickel, et al. 2025) suggested differently, as we found decreased MMR amplitudes (i.e., more negative, mature MMRs) in older children with DLD, but increased MMR amplitudes (i.e., more positive, less mature MMRs) in older TD children (Themas, Nickel, et al. 2025). This discrepancy to the findings of this longitudinal study might be explained by observations suggesting the maturational trajectory of the MMR to not be linear (Werwach et al. 2022). It could be that the group of TD children have gone through a change toward positivity that was measured at T1 and then started to increase again resulting in a negative MMR at T2. Even if the latter indicates that during childhood 1 year between repeated measures might be too long of a period to capture nuances in the maturational trajectory of the MMR, the longitudinal approach used in the current study is superior and more robust compared to single measurements, allowing us to draw more general conclusions, namely, that the amplitude of the MMR became more mature in both groups.

In contrast to our findings on auditory duration discrimination (durQ1), the cluster‐based permutation tests showed no significant deviant‐standard differences in the pitchdurQ3 condition. We would have expected the MMR to mature from T1, where we also did not find any significant MMRs in this condition, to T2, as, for example, Wu et al. 2025 found an adult like MMN in similarly aged children in response to pitch contour changes. However, in contrast to Wu et al. (2025)'s stimuli, our deviant differed by two, not only one feature from the standard. Even though it has been argued that auditory features are processed by independent neural mechanisms, such that the brain's response to a deviant differing by two features from the standard should approximately be equal to the sum of each individual response (Paavilainen et al. 2001, also see Ylinen et al. 2005), for the maturing brain it might be difficult to discriminate two auditory features together. Another factor that should have facilitated the perception of pitch contour and duration is that these features were presented within a meaningful and highly frequent Estonian word. Studies have shown stronger MMN in adults (Zora et al. 2015) and more mature MMN in 4‐ to 5‐year‐old children (Campos et al. 2023) when phonological changes (e.g., in prosody) are perceived in meaningful words compared to pseudowords. Additionally, word frequency in a language also plays a role, such that high‐frequency words are processed more efficiently by both adults (Alexandrov et al. 2011; Aleksandrov et al. 2017) and 3‐year‐old children (Strotseva‐Feinschmidt et al. 2015). Taken these results into account, the pitchdurQ3 condition deviant should have elicited an MMN as it occurs more frequently (563.49 per million tokens; Institute of the Estonian Language 2023) in Estonian than the durQ1 deviant (29.18 per million tokens; Institute of the Estonian Language 2023). As, however, only the durQ1 deviant elicited an MMR, the discrimination of the two prosodic cues, pitch contour and duration, in the pitchdurQ3 condition appears to have posed a challenge for participants independent of word frequency.

Finally, it should also be considered that the underlying effect could be small and difficult to detect given our relatively small sample size and the inherent noisiness and individual variability of child EEG data. Further investigation is warranted to determine the factors underlying the potential absence of the MMR in response to pitch contour and duration in the current study.

### 4.2 Group Differences in the MMRs

As we did not find any MMRs in the pitchdurQ3 condition, group differences could only be analyzed for the durQ1 condition. We expected significant group differences in the MMR amplitude with less pronounced maturation of the MMR in children with DLD over the course of 1 year, reflected in a less negative MMR at the second measuring time point (Männel et al. 2017; Schaadt and Männel 2019). Even though we did not find group differences at T1, we found significant group differences in the negative cluster at T2, with a less pronounced negative MMR in children with DLD (see also Chen et al. 2016; Choudhury and Benasich 2011). Furthermore, we found differences in the maturational patterns between the two groups, with a significant longitudinal change observed in the TD group but not in the DLD group. This pattern of results may indicate that MMR maturation in TD children follows a more stable and robust trajectory, while the absence of consistent change in the DLD group may point to greater variability of maturational pathways among children with DLD. This proposed variability in maturation aligns with accounts of DLD emphasizing substantial heterogeneity within the population, whereby language difficulties arise from differing combinations of underlying cognitive deficits rather than a single impairment (Tomas and Vissers 2019). Thus, as children with DLD might rely on different neural or cognitive mechanisms during auditory processing, a consistent maturational pattern in their MMR was absent. The heterogeneity in the DLD group also might explain the nonsignificant differences in the maturational trajectories of the MMR between groups because it obscures consistent group‐level differences.

In addition to variability in maturational trajectories, the present findings might point to a general delay in the maturation of auditory processing in children with DLD. Although the estimated trajectories suggested a similar overall direction of change across groups (see Figure 5), the absence of a significant longitudinal change in the DLD group, together with greater variability in individual trajectories, may indicate that maturational processes occur more slowly or less consistently in this population. This interpretation aligns with the immature auditory processing account of DLD, which proposes a delay in cortical auditory development (Bishop and McArthur 2004; Bishop and McArthur 2005; McArthur and Bishop 2004; Kwok et al. 2018). For example, Kwok et al. (2018) reported no overall group differences in auditory evoked potential (AEP) maturity between 7‐ and 10‐year‐old children with DLD and TD peers but demonstrated that children with moderate–severe DLD exhibited delayed neural responses relative to their chronological age. Importantly, these effects were only evident when considering subgroups based on severity, suggesting that maturational delay may be present only in a subset of children with DLD. In the present study, the lack of stratification by severity may therefore have contributed to the absence of a clear group‐level difference in the observed longitudinal change.

Finally, the use of meaningful stimuli must be considered when interpreting the present results. The less negative MMR at T2 and the absence of a statistically reliable group difference in longitudinal change may reflect not only difficulties in auditory discrimination, but also differences in language competence in the DLD group. As discussed by Bishop (2007), the MMN/MMR is sensitive to experience‐dependent factors and may be influenced by the quality of phonological representations in long‐term memory. Specifically, MMN amplitudes have been shown to be larger for stimuli that have established representations in the mental lexicon compared to novel or less familiar stimuli. Consistent with this view, several studies have demonstrated enhanced MMN responses to linguistically meaningful stimuli (e.g., Aleksandrov et al. 2017; Hasting et al. 2008; Pettigrew et al. 2004; Pulvermüller et al. 2001; Zora et al. 2015, 2016, 2023). Within this framework, the reduced negativity observed in the DLD group may reflect less robust phonological and lexical representations of the stimuli, which may also influence the efficiency of auditory discrimination processes. Thus, diminished MMR amplitudes should be interpreted with caution, as they may reflect differences in language‐related representations when processing meaningful words, rather than purely auditory discrimination processes (Bishop et al. 2012). Importantly, these findings demonstrate that differences between TD and DLD children emerge in linguistically meaningful contexts.

### 4.3 Relationship Between Neurophysiological and Behavioral Discrimination

Similar to the maturation of the MMR to the durQ1 condition, behavioral results showed increased accuracy and decreased reaction times at T2 compared to T1 when quantity degrees had to be discriminated. Further and in line with the neurophysiological data showing MMRs in the durQ1, but not in the pitchdurQ3 condition, the behavioral results also suggest duration changes to be easier to detect (discrimination of Q1 vs. Q2) compared to duration and pitch changes (discrimination of Q2 vs. Q3). Finally, we found children with DLD to perform less accurately in the behavioral tasks compared to their TD peers, which was also represented in the neurophysiological data as the results show a less negative MMR and possibly slower maturation in the durQ1 condition in the DLD group compared to their TD peers.

To further tackle the relationship between neurophysiological and behavioral auditory discrimination, we also analyzed the associations between the change in the MMR amplitude from T1 to T2 and the *d*′ scores of the behavioral tasks at T1 and T2 at the individual level. MMR amplitude was not significantly related to any behavioral discrimination score in the tested models. This is in line with several studies that also have not found correspondence between behavioral‐neurophysiological discrimination (Bishop et al. 2010; Halliday et al. 2014; McArthur and Bishop 2005; Nan et al. 2018; Pihko et al. 2006; Uwer et al. 2002). This may reflect differences in task demands between neurophysiological and behavioral discrimination. As such, behavioral discrimination measures additional abilities beyond auditory discrimination, and the ability to behaviorally discriminate auditory features is influenced by these extra demands—especially for children and more so for children with DLD. One may therefore assume that in adult studies the relationship between the MMN amplitude and behavioral auditory discrimination is more consistently found as behavioral task demands do not influence adults as much as children and therefore allow for a more precise estimation of behavioral auditory discrimination. However, contradictory findings are also reported in the adult literature (Näätänen et al. 2014; Pakarinen et al. 2007; Zhao 2022) and the neurophysiological‐behavioral relationships at individual level are seldom reported (Zhao 2022). Näätänen et al. (2007) attributed the lack of associations in adult studies to the fact that MMN tends to be replicable at the group level but considerably less so at the individual level.

Furthermore, the relationship between MMR and behavioral measures is likely more complex and influenced by developmental factors, such that simple linear approaches may be insufficient to capture it (Ahmmed et al. 2008; Clarke and Adams 2007). Consistent with this view, some studies have applied more sophisticated analytical approaches to examine the relationship between ERPs and behavioral performance. Bishop et al. (2012) researched causal links between T‐complex (an ERP recorded at temporal scalp sites, reflecting activity in the secondary auditory cortex) and language impairment with structural equational models and found that atypical ERP responses in children with DLD are better explained as a downstream consequence of phonological and language difficulties, rather than as an endophenotypic mediator of genetic risk. Furthermore, Partanen et al. (2022) examined associations between musical training, perceptual musical skills, auditory processing indexed by the MMN, and reading ability using structural equation modelling. They found that performance on perceptual musical tasks was more directly associated with reading skills than indirectly mediated through low‐level auditory discrimination abilities as indexed by the MMN. The results of Bishop et al. (2012) and Partanen et al. (2022) are consistent with the present findings, which likewise did not reveal evidence for a direct association between atypical MMR responses and behavioral discrimination performance. Rather and in line with Partanen et al.'s (2022) explanation it could be that the absence of behavioral‐MMR associations in the present study is not unexpected, as neurophysiological auditory measures may only relate to behavioral performance once processing is sufficiently impaired (see also Halliday and Bishop 2005). Overall, consistent with these previous findings, the current results suggest that the neurophysiological‐behavioral relationship is complex and was not directly observed in the present data.

### 4.4 Limitations

As already noted, the conclusions of the present study are based on a relatively small sample size, reflecting difficulties in recruiting participants with DLD who met strict diagnostic criteria in a country with a small and geographically dispersed population, such as Estonia, especially during the COVID‐19 pandemic. The relatively limited sample size and high interindividual variability, especially within the DLD group, may have reduced statistical power, thereby lowering the likelihood of detecting reliable differences in longitudinal trajectories of the MMR. Additionally, the comparison of time slopes across groups aggregates variability within each group, which may obscure existing differences at the individual level. Therefore, the nonsignificant findings in the MMR analyses should be interpreted with caution. Future studies with larger samples, particularly within the DLD population, are needed to more reliably characterize developmental patterns in MMR maturation.

Related to the limited sample size, we did not perform a priori power analysis, even though we acknowledge its importance for avoiding Type II error. However, due to the high specificity of the stimuli used in the present study and as the properties of the MMR are highly dependent on the nature of the stimuli (e.g., Themas et al. 2023), extracting an effect size from studies using different stimuli or study populations, would have led to an erroneous estimation of the effect size. Given that our study was the first EEG study conducted with Estonian children, performing a power analysis would have required speculation, potentially leading to inaccurate conclusions about the required sample size.

The age range of the participants was relatively broad, given the overall sample size. This choice was primarily driven by the recruitment challenges described above. Previous findings, including our own data (Themas 2025) and other research (Werwach et al. 2022), suggest that maturational changes in MMR amplitude may be nonlinear and can occur over relatively short time periods. Therefore, the wide age range may be considered a limitation, as it could introduce additional heterogeneity into the data. Specifically, differently aged children may exhibit distinct phases of MMR maturation, such that younger participants may show increased positivity, whereas older participants may begin to display emerging negative responses.

At the same time, the broad age range also offers certain advantages. It allows age to be treated as a continuous variable, enabling a more fine‐grained examination of maturational changes that better reflect their continuous nature. This approach is consistent with the view that development is gradual rather than occurring in discrete stages and thus treating age as a continuous variable avoids imposing artificial boundaries between age groups. Overall, future research should carefully consider the choice of age range and determine whether grouping participants or modelling age as a continuous variable is more appropriate for capturing developmental processes.

## 5 Conclusions

In conclusion, our findings suggest that auditory discrimination of duration changes within the Estonian three‐way quantity system, measured by using the MMR, is still maturing at preschool age. The absence of significant longitudinal change in children with DLD may indicate delayed and/or more variable maturation of auditory processing in response to quantity degree contrasts. No reliable MMN responses were observed in the combined pitch contour and duration change processing either at T1 or at T2, which may indicate that the distinction in question still poses a challenge at late preschool and early school age. Although this represents a novel and important finding, as the perception of the combination of these prosodic features has not been reported in the major languages studied to date, further research is warranted to verify these results. Additionally, no significant associations were identified between changes in MMR amplitude and performance in the behavioral discrimination tasks, which show that the relationship is likely more complex and influenced by additional developmental factors that were not directly measured in the present study.

Even though our findings should be interpreted with caution due to the limited sample size and the inclusion of only two longitudinal measurement points, our study offers valuable insights into the maturation of auditory change detection mechanisms and contributes to a deeper understanding of how neurophysiological and behavioral measures interact in both TD and language‐impaired Estonian children.

## Author Contributions

**Liis Themas:** conceptualization, investigation, writing – original draft, methodology, visualization, formal analysis, project administration, data curation. **Gesa Schaadt:** conceptualization, writing – original draft, methodology, validation, visualization, supervision. **Pärtel Lippus:** methodology, writing – review and editing, supervision, conceptualization, validation. **Marika Padrik:** methodology, writing – review and editing, supervision, conceptualization, validation. **Liis Kask:** investigation, writing – review and editing, resources, validation. **Ulvi Vaher:** investigation, writing – review and editing, resources, validation. **Mairi Männamaa:** investigation, writing – review and editing, resources, validation. **Kairi Kreegipuu:** conceptualization, funding acquisition, methodology, validation, writing – review and editing, supervision, resources.

## Funding

This work was supported by the Eesti Teadusagentuur (PRG1151) and the Estonian Center of Excellence for Well‐Being Sciences (TK218).

## Disclosure

During the preparation of this work the author(s) used ChatGPT Version 4 in order to correct the use of English language. After using this tool, the authors reviewed and edited the content as needed and take full responsibility for the content of the publication. Additionally, ChatGPT was used to find mistakes in code and generate some parts of code, which were used for data analysis. Before use, the generated code was reviewed by the authors.

## Conflicts of Interest

The authors declare no conflicts of interest.

## Supporting information


**Table 1A.** Nouns and corresponding pseudowords used in the second behavioral task.
**Figure 1B.** Contrasts of the responses to the deviant and standard stimuli based on the cluster‐based permutation tests across both groups (children with and without DLD) in durQ1 condition at T1. White asterisks on the topographic plots indicate significant differences between the response to the standard and deviant stimuli.
**Figure 2B.** Contrasts of the responses to the deviant and standard stimuli based on the cluster‐based permutation tests across both groups (children with and without DLD) in pitchdurQ3 condition at T1. White asterisks on the topographic plots indicate significant differences between the response to the standard and deviant stimuli.
**Figure 3B.** Contrasts of the responses to the deviant and standard stimuli based on the cluster‐based permutation tests across both groups (children with and without DLD) in durQ1 condition at T2. White asterisks on the topographic plots indicate significant differences between the response to the standard and deviant stimuli.
**Figure 4B.** Contrasts of the responses to the deviant and standard stimuli based on the cluster‐based permutation tests across both groups (children with and without DLD) in pitchdurQ3 condition at T2. White asterisks on the topographic plots indicate significant differences between the response to the standard and deviant stimuli.

## Data Availability

The raw EEG data that support the findings of this study are available on request from the corresponding author in University of Tartu Library DataDOI repository at https://doi.org/10.23673/re‐525. The data are not publicly available due to privacy or ethical restrictions. The aggregated EEG data and codes that support the findings of this study are openly available in Zenodo repository at https://doi.org/10.5281/zenodo.15799644.

## Endnotes

^1^
Compared with the preregistration, the goals and hypotheses presented in the paper have been refined to provide a clearer focus in line with the reviewers' recommendations. However, the fundamental aim of the study—and, critically, the materials and methods—remain unchanged.
