# Supplementary material for: Mismatch Responses to Prosodic Changes in Children With Developmental Language Disorder: A Longitudinal Approach
Source: Eur J Neurosci. 2026 Oct 12;64(7):e70710. doi: 10.1111/ejn.70710 (PMC13659036; doi:10.1111/ejn.70710)
Supplement: Supplementary file 1 [file EJN-64-0-s001.pdf]

Appendices

Appendix A – Stimuli of the lexical decision task

Table 1A. Nouns and corresponding pseudowords used in the second behavioral task

Nouns	Transcription	Meaning	Correct Q	Pseudoword	Changed Q
pudel	[putel]	*bottle*	Q1	[pu:tel]	Q2
sibul	[sipul]	*onion*	Q1	[si::pul]	Q3
tomat	[tomɑt:]	*tomato*	Q1	[to:mɑt:]	Q2
jänes	[jænes]	*rabbit*	Q1	[jæ::nes]	Q3
redel	[retel]	*ladder*	Q1	[re:tel]	Q2
küünal	[ky:nɑl]	*candle*	Q2	[ky::nɑl]	Q3
saabas	[sɑ:pɑs]	*boot*	Q2	[sɑ::pɑs]	Q3
seelik	[se:lik:]	*skirt*	Q2	[selik:]	Q1
käärid	[kæ:rit]	*scissors*	Q2	[kærit]	Q1
raamat	[rɑ:mɑt:]	*book*	Q2	[rɑ::mɑt:]	Q3
diivan	[ti::vɑn]	*couch*	Q3	[tivɑn]	Q1
röövel	[rø::vel]	*robber*	Q3	[rø:vel]	Q2
peegel	[pe::kel]	*mirror*	Q3	[pekel]	Q1
tiiger	[ti::ker]	*tiger*	Q3	[tiker]	Q1
kiiver	[ki::ver]	*helmet*	Q3	[ki:ver]	Q2

Note. Nouns – the 15 nouns used in the second behavioral task; Transcription – IPA transcription of the nouns; Meaning – translation of the nouns from Estonian to English; Correct Q – the quantity degree of the nouns; Pseudoword – IPA transcription of the corresponding pseudoword created from the noun; Changed Q – the quantity degree of the pseudoword.

Appendix B – Figures of the cluster-based permutation test results

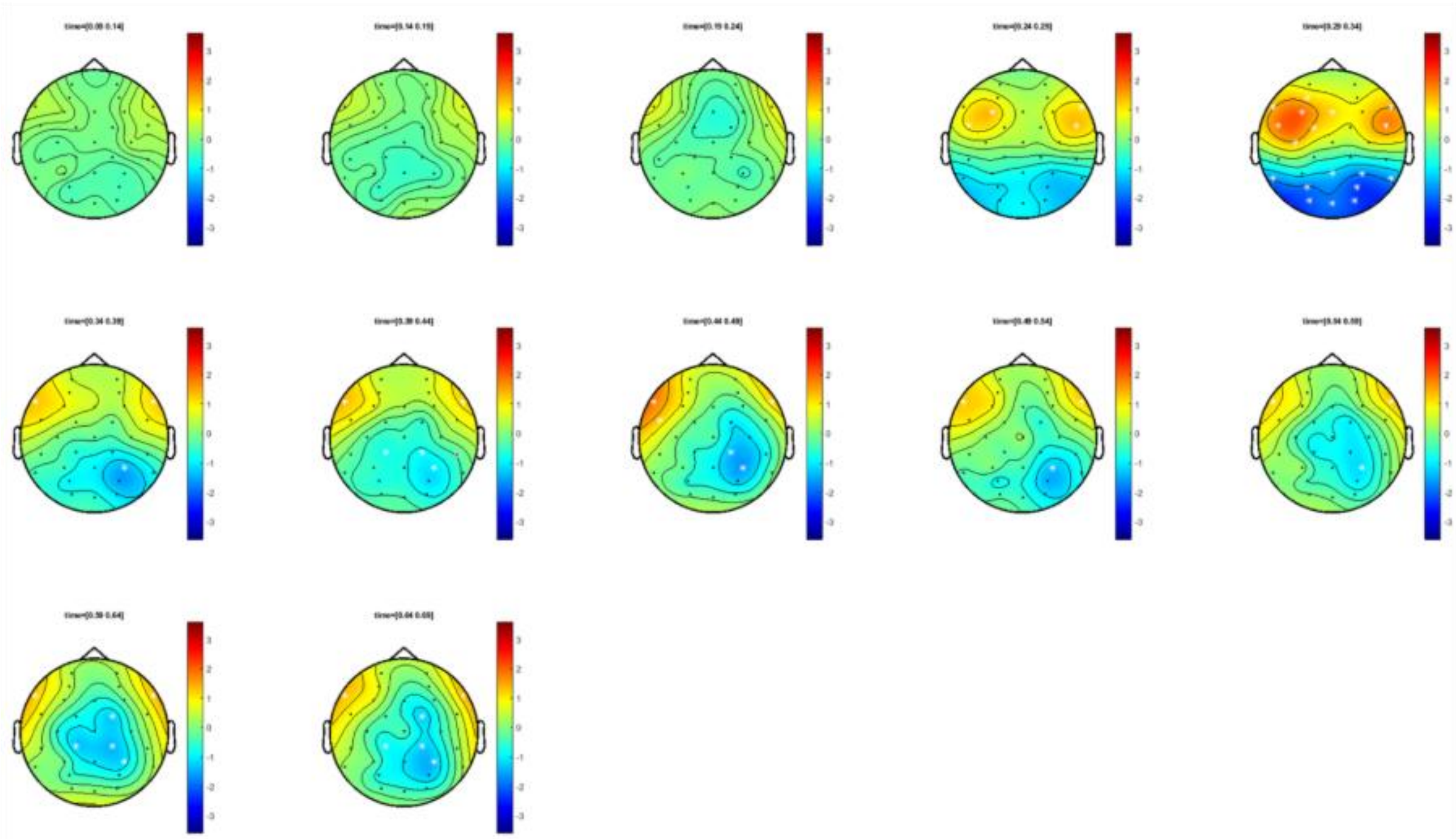

Figure 1B. Contrasts of the responses to the deviant and standard stimuli based on the cluster-based permutation tests across both groups (children with and without DLD) in durQ1 condition at T1. White asterisks on the topographic plots indicate significant differences between the response to the standard and deviant stimuli.

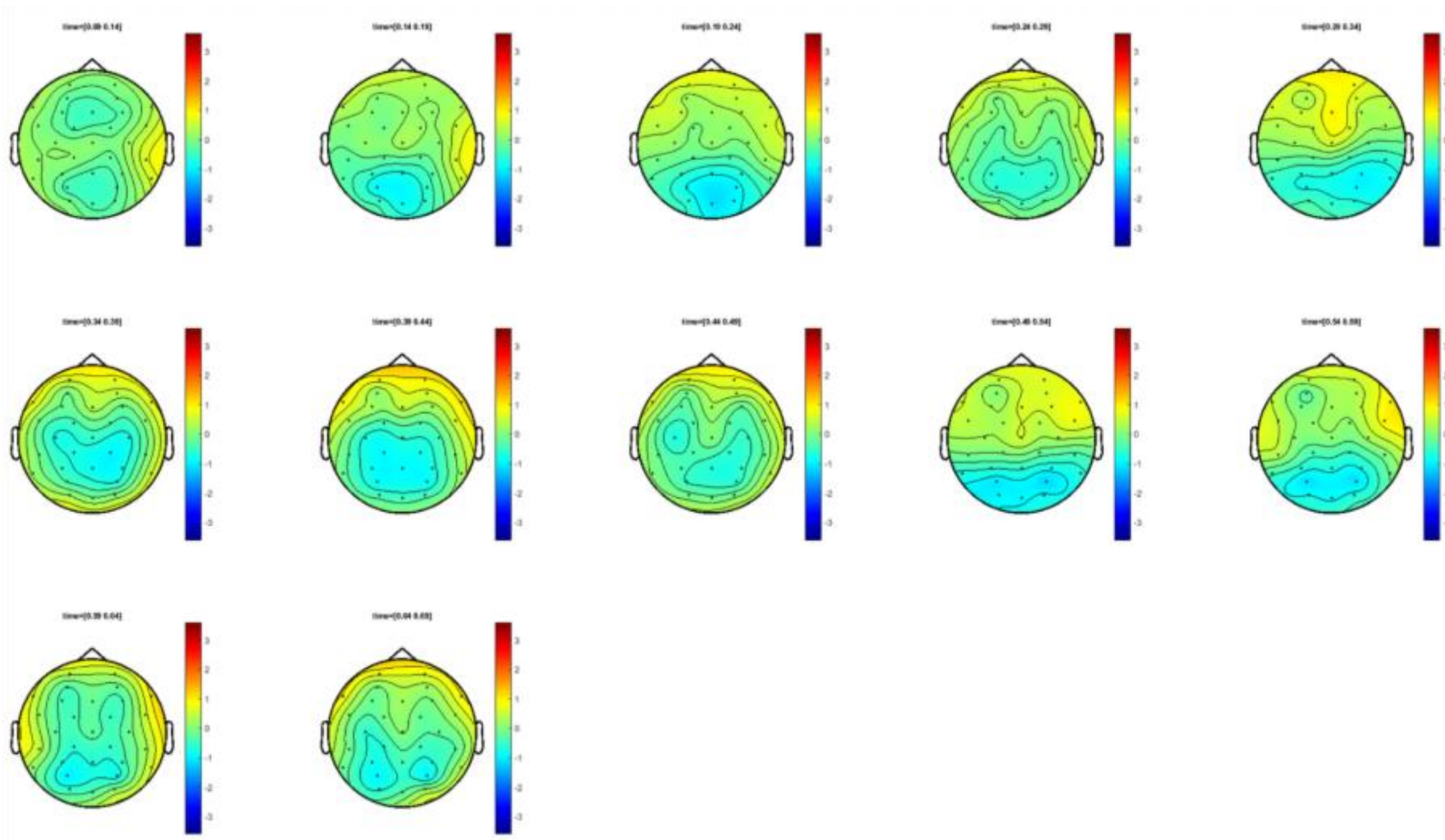

Figure 2B. Contrasts of the responses to the deviant and standard stimuli based on the cluster-based permutation tests across both groups (children with and without DLD) in pitchdurQ3 condition at T1. White asterisks on the topographic plots indicate significant differences between the response to the standard and deviant stimuli.

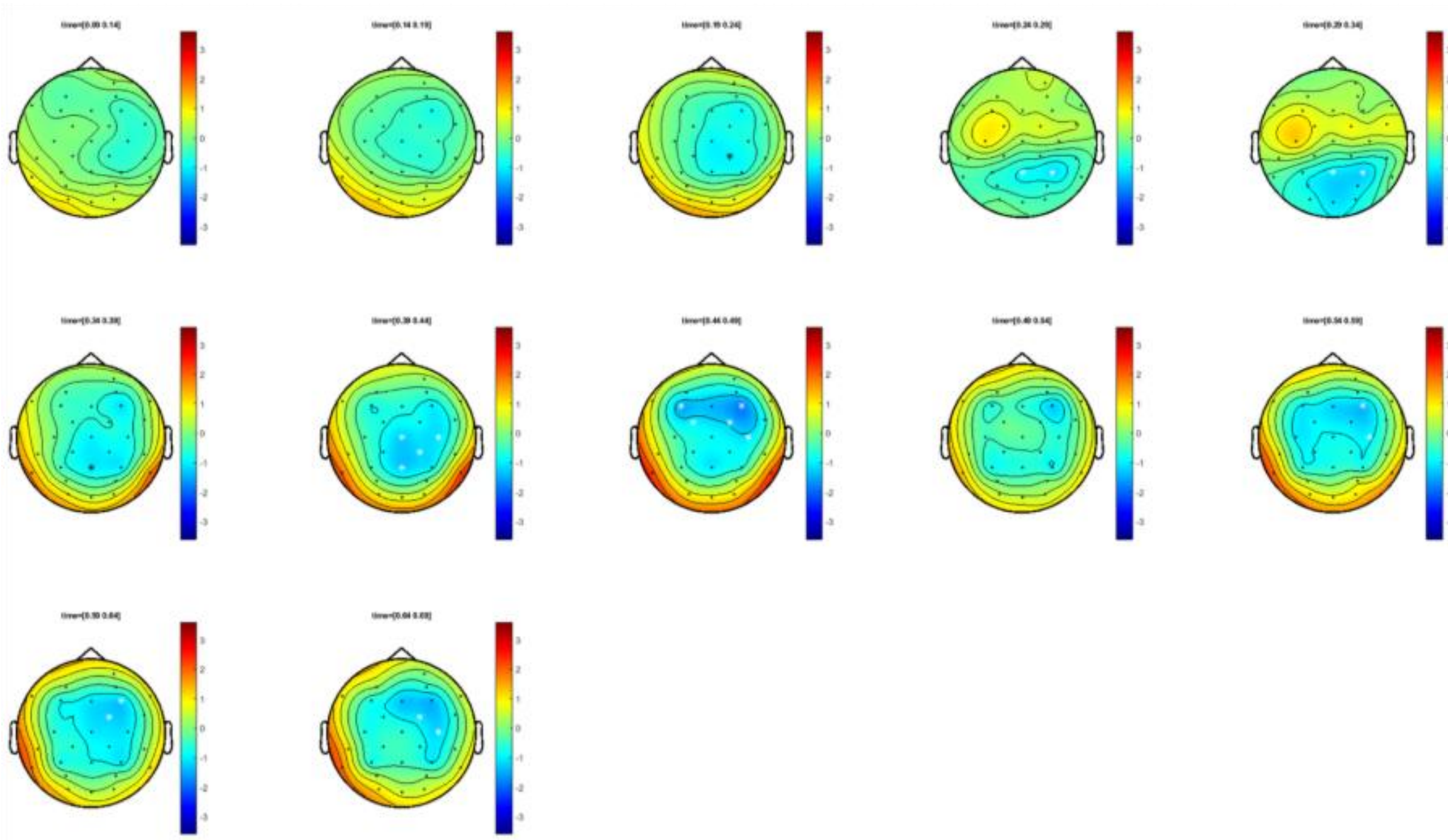

Figure 3B. Contrasts of the responses to the deviant and standard stimuli based on the cluster-based permutation tests across both groups (children with and without DLD) in durQ1 condition at T2. White asterisks on the topographic plots indicate significant differences between the response to the standard and deviant stimuli. ‘

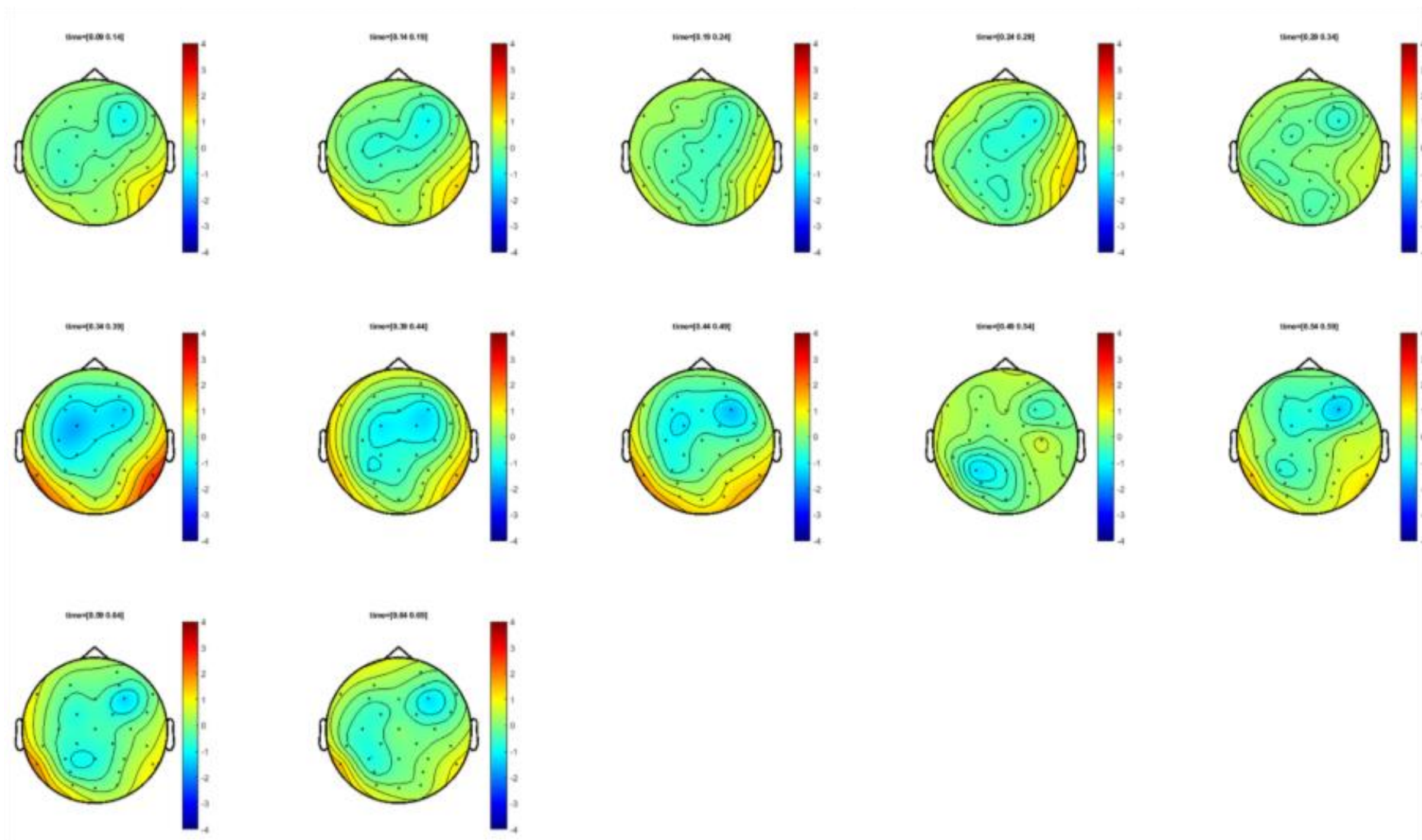

Figure 4B. Contrasts of the responses to the deviant and standard stimuli based on the cluster-based permutation tests across both groups (children with and without DLD) in pitchdurQ3 condition at T2. White asterisks on the topographic plots indicate significant differences between the response to the standard and deviant stimuli.

Appendix C – Additional materials for the cluster-based permutation test results for duration discrimination

At T1, an increased negativity in response to the deviant stimulus was observed at occipital and parietal sites (O1, Oz, O2, PO3, PO4, P7, P8, Pz, P4) between 290–340 ms post-stimulus. This was followed by a sustained positive shift at frontal sites (F7 and F8) between 340 to 690 ms after stimulus onset. Concurrently, a more negative deflection emerged in the deviant condition at a few centro-parietal electrodes (P4, CP1, CP2), culminating in a broader right-lateralized cluster (FC2, CP2, P4, CP1) between 590 and 690 ms.

At T2 two smaller clusters were found: 1) 240-340 ms after stimulus onset at Pz and P2; 2) 540-690 ms after stimulus onset at F4, C4, FC2.
